# Association between Physical Fitness and Cardiovascular Health in Firefighters

**DOI:** 10.3390/ijerph20115930

**Published:** 2023-05-23

**Authors:** Jaron Ras, Denise L. Smith, Elpidoforos S. Soteriades, Andre P. Kengne, Lloyd Leach

**Affiliations:** 1 Department of Sport, Recreation and Exercise Science, Faculty of Community and Health Sciences, University of the Western Cape, Cape Town 7535, South Africa; lleach@uwc.ac.za; 2 Health and Human Physiological Sciences, Skidmore College, Saratoga Springs, NY 12866, USA; dsmith@skidmore.edu; 3 Healthcare Management Program, School of Economics and Management, Open University of Cyprus, Nicosia 2220, Cyprus; elpidoforos.soteriades@ouc.ac.cy; 4 Harvard T.H. Chan School of Public Health, Department of Environmental Health, Environmental and Occupational Medicine and Epidemiology (EOME), Boston, MA 02115, USA; 5 Non-Communicable Diseases Research Unit, South African Medical Research Council, Cape Town 7505, South Africa; andre.kengne@mrc.ac.za

**Keywords:** firefighters, physical fitness, risk factor, cardiorespiratory, strength, endurance, cardiovascular health

## Abstract

Firefighters perform strenuous work in dangerous and unpredictable environments requiring optimal physical conditioning. The aim of this study was to investigate the association between physical fitness and cardiovascular health (CVH) in firefighters. This cross-sectional study systematically recruited 309 full-time male and female firefighters between the ages of 20 to 65 years in Cape Town, South Africa. Physical fitness was assessed using absolute (abV̇O_2max_) and relative oxygen consumption (relVO_2max_), grip and leg strength, push-ups and sit-ups, sit-and-reach for flexibility and lean body mass (LBM). CVH encompassed age, smoking, blood pressure (BP), blood glucose, lipid profile, body mass index, body fat percentage (BF%), and waist circumference. Linear regressions and logistic regressions were applied. Multivariable analysis indicated that relVO_2max_ was associated with systolic BP (*p* < 0.001), diastolic BP (*p* < 0.001), non-fasting blood glucose (*p* < 0.001), and total cholesterol (*p* = 0.037). Poor CVH index was negatively associated with relV̇O_2max_ (*p* < 0.001), leg strength (*p* = 0.019), and push-ups (*p* = 0.012). Furthermore, age was inversely associated with V̇O_2max_ (*p* < 0.001), push-up and sit-up capacity (*p* < 0.001), and sit-and-reach (*p* < 0.001). BF% was negatively associated with abV̇O_2max_ (*p* < 0.001), grip and leg strength (*p* < 0.001), push-ups (*p* = 0.008), sit-ups (*p* < 0.001), and LBM (*p* < 0.001). Cardiorespiratory fitness, muscular strength, and muscular endurance were significantly associated with a better overall CVH profile.

## 1. Introduction

Firefighters routinely perform strenuous work in dangerous and unpredictable environments [1,2]. This requires firefighters to wear heavy, insulated personal protective equipment (PPE) that places additional strain on their bodies [2,3,4]. This necessitates that firefighters maintain all aspects of their physical fitness to cope with such stressors. Research has shown that in the first three minutes before the arrival to an alarm response, energy output increases by 400 to 600% [5]. While performing their duties, firefighters rapidly reach near-maximum heart rates and are, often, required to sustain these levels for prolonged periods of time [6,7]. 

It is well documented that close to 50% of all firefighting-related deaths are attributed to underlying CVD risk factors, with the majority of these deaths occurring during or shortly after fire suppression [8]. It has also been shown that there is an increase of up to 20.9% in metabolic rate while firefighters wear PPE [9]. Several firefighting tasks average an oxygen consumption (V̇O_2_) of 26.1 to 30.4 mL·kg·min, with the most strenuous tasks requiring an average of 44.0 mL·kg·min [10]. Firefighters were reported to spend the least time on fire suppression and fireground activities [11], but these duties resulted in the highest energy requirement and highest incidence of cardiovascular incidents [1]. Research suggests that firefighters should maintain a cardiorespiratory fitness level of 42 mL·kg·min to manage these job stressors adequately [12]. The metabolic demands of firefighting highlight the need for firefighters to maintain an adequate level of physical fitness in order to perform their duties aptly [9,10]. Many firefighting tasks require firefighters to perform multiple forceful repetitions and/or maintain strong isometric contractions, such as during forcible entry, equipment carries, equipment hoist and hose drag [13,14,15]. Moreover, the higher the force produced by firefighters, the more efficiently firefighters can perform their duties [13,14,15]. Generally, more fit and stronger firefighters would participate in regular physical activity, which is crucial in maintaining adequate levels of physical fitness, which is particularly important as firefighters age [2,16,17]. It has been documented that firefighters are aware of the necessity of maintaining adequate levels of physical fitness and the benefit of remaining physically active [18,19]. However, many firefighters reported developing poor attitudes toward their health, particularly as they age and remained longer in the profession [20,21]. Ageing has also been related to a progressive decrease in cardiorespiratory fitness, muscular strength and endurance in firefighters [22,23,24,25]. In addition, ageing has been related to the gradual decrease in CVH [26,27,28], making this population particularly vulnerable when performing fire and rescue operations. 

The association between physical fitness and CVH is bidirectional, where physical fitness influences CVH and vice versa [7,29,30,31,32,33]. Firefighters that are fitter and stronger with higher muscular strength were found to have a more favourable body composition, and better overall CVH profile [30,31,32,34]. Most studies on firefighters have been conducted in the United States, Canada, Europe and Australia [35]. However, this association has not been studied among firefighters on the African continent. Previous studies have shown that firefighters, in South Africa, have a high prevalence of CVD risk factors, particularly those related to obesity, hypertension and physical inactivity [18,26,36], with this population having a poor attitude toward physical activity and physical fitness [18,20]. This suggests that this population likely have a poor level of physical fitness and CVH compared to other firefighting populations previously mentioned, which, generally, had a, comparatively, healthier firefighting population [28,31,37]. However, more research is needed assessing the CVH of firefighters in South Africa, where very little research exists. In addition, because the population of firefighters where the association between physical fitness and CVH had been assessed were, generally, healthier, it is expected that the current study may provide unique outcomes in comparison. The absence of research on the African firefighter population likely contributes toward the paucity of policy regulations compared to the aforementioned nations, particularly on firefighters maintaining minimum physical fitness and cardiovascular health standards aimed at ensuring the physical well-being of firefighters. There have not been any previous studies investigating the association between measures of physical fitness and CVH, and the literature contains little information about CVH index (CVHI) in firefighters. In addition, the findings of this study will highlight the importance of physical fitness in maintaining CVH and vice versa in firefighters. Therefore, the aim of this study was to investigate the association between physical fitness and CVH in firefighters in South Africa. 

## 2. Materials and Methods

### 2.1. Study Design and Population

In this study, we used a quantitative, non-experimental, cross-sectional research design by collecting data on physical fitness (cardiorespiratory fitness, muscular strength and endurance, flexibility, and body composition), CVH [cardiovascular disease (CVD) risk factors, CVD risk score, heart rate variability (HRV), and CVH index (CVHI)] in firefighters. This study was implemented between June and August 2022. Each volunteer participant provided written informed consent. In total, 309 full-time male and female firefighters between the ages of 20 to 65 years from the CoCTFRS participated in this study. Ethical approval was granted by the Biomedical Research Ethics Committee (ethical clearance number: BM21/10/9) of the University of the Western Cape. Approval was also granted by the Chief Fire Officer as well as the Department of Policy and Strategy research branch of the City of Cape Town. 

### 2.2. Sampling and Participant Recruitment

Data collection took place during the annual physical fitness assessment conducted by the CoCTFRS at a standardised fire station located in the City of Cape Town metropolitan area. Systematic random sampling was used to select firefighters to participate in this study, where every third firefighter was selected to participate from 96 platoons (32 fire stations). Each of the 96 platoons consisted of 8 to 12 firefighters. All full-time firefighters between the age range of 20–65 years were recruited to participate. Firefighters were excluded from participation if they were on administration duty, on sick leave, employed on a part-time or seasonal basis, and did not participate in the physical ability test (PAT) on the day of testing. 

### 2.3. Descriptive Measures

Data were collected using a researcher-generated questionnaire retrieving information on firefighters’ sociodemographic and lifestyle information. Descriptive measures were objectively measured by trained researchers [38] and included height, weight, body fat percentage (BF%), lean body mass (LBM), blood pressure, non-fasting blood glucose (NFBG) concentration, blood cholesterol concentrations, and heart rate variability (HRV). 

Briefly, for stature, firefighters were asked to stand barefoot on the level stadiometer base, with the heels together and the heels, buttocks, and upper back touching the stadiometer rod of a portable stadiometer (Seca model 700, Gmbh & Co., Hamburg, Germany). Body mass, body fat percentage and lean body mass were obtained using a Tanita© (Tanita©, Tokyo, Japan) BC-1000 Plus bioelectrical impedance (BIA) analyser. When taking body mass and BF%, firefighters were requested to wear minimal clothing and to stand barefoot, upright, with feet apart. Waist circumference was measured at the point of the belly button [39]. Hip circumference was taken at the level of the greatest posterior protuberance of the buttocks [39]. Blood pressure was taken thrice using an Omron Healthcare, Inc. M6 comfort intelligence (Omron Healthcare Co., Ltd., Hoofddorp, The Netherlands) automatic blood pressure monitor, with at least two-minute intervals between measures. Blood measures were measured using a CardioChek^®^ Plus analyser (PTS Diagnostics, Indiana, IN, USA), using the finger prick method and a standard pipet. A detailed explanation of the methods used to conduct each test and the cut-offs for each CVD risk factor can be found at Ras et al. [38] (https://doi.org/10.3390/ejihpe12110120, accessed on 10 February 2023).

### 2.4. Physical Fitness Measures

#### 2.4.1. Cardiorespiratory Fitness

Due to concerns about the possibility that a maximum exercise stress test would cause undue fatigue in firefighters, who were also required to return to shift after testing and perform firefighting duties, cardiorespiratory fitness was estimated using the non-exercise method via the following formula: V̇O_2max_ = 3.542 + (−0.014 × Age) + (0.015 × Body Mass [kg]) + (−0.011 × Resting Heart Rate) [40]. Two measures were used for cardiorespiratory fitness, namely absolute V̇O_2max_ (abV̇O_2max_) and relative V̇O_2max_ (relV̇O_2max_).

#### 2.4.2. Handgrip Strength

Using a Takei® 5401-C handgrip dynamometer, handgrip strength was used to assess upper body muscular strength using the standardized procedures recommended by the American College of Sports Medicine (ACSM) [39]. To ensure that the second phalangeal joint fits comfortably under the handle. To ensure consistency, firefighters were requested to hold the handgrip dynamometer in line with the forearm and the level of the thigh and away from the body. If firefighters flexed their elbow joint or moved the dynamometer significantly from the starting position, the firefighter was asked to repeat the measure. The dynamometer was set to zero, and, thereafter, firefighters were asked to squeeze with as much force as possible without holding their breath. The procedure was repeated twice, and the highest reading of the two measures was recorded. Manufacturer accuracy for the handgrip is ±2.0 kg force (kgf).

#### 2.4.3. Leg Strength Dynamometer

Leg strength was measured with a Takei^®^ back and leg strength dynamometer. Firefighters were requested to remain upright, with their feet placed shoulder width apart, on the base of the dynamometers. With their palms in the prone position, firefighters were requested to maintain an extended position in their elbow joints while their hands grasped the dynamometer hand bar. The chain was adjusted to the midpoint of the patella tendon to ensure that each firefighter’s knees were in approximately 110 degrees of flexion. The firefighters were instructed to pull as forcefully as possible on the chain while attempting to straighten their knees. The procedure was repeated twice, and the highest reading of the two was recorded. Manufacturer accuracy for the back and leg strength dynamometer were ±6.0 kgf, respectively.

#### 2.4.4. Push-Ups

Upper body muscular endurance was assessed using the push-ups test [39] and conducted in accordance with the ACSM guidelines [39]. Males were requested to position themselves in the standard prone position, with their hands positioned under the shoulder joint, back in a straight position and level position, in line with the head and their toes serving as the pivotal point. Females were requested to perform the modified push-up position, with their hands positioned under the shoulder joint, back straight and in line with the head, legs together, with their lower leg in contact with the mat, ankles placed in a planter-flexed position, and their knees acting as the pivotal point. For each repetition, firefighters were required to fully raise their body off the mat extending their elbow joints and returning to the down position. A hedgehog was placed under the chest of firefighters to maintain consistency when counting each repetition. The test was stopped when firefighters could not perform an additional push-up or two consecutive push-ups were performed incorrectly.

#### 2.4.5. Sit-Ups

The sit-ups test was used to assess abdominal muscular endurance [39]. The firefighters were requested to lie down in a supine position on the mat with their knees at 90 degrees flexion, with their hands across the shoulders and elbows pointing forward [41]. For each repetition, firefighters were required to touch their knees with their elbows and then go back to the starting position, ensuring their shoulders touch the floor. The number of repetitions performed in 60 s was recorded. The test ended when one minute had passed or the firefighters’ experienced exhaustion, which presented as the inability to perform another repetition [40].

#### 2.4.6. Flexibility

To assess lower back and hamstring flexibility, the sit-and-reach method was used and conducted in accordance with the guidelines recommended by the ACSM [39]. The firefighters were asked to position themselves in a seated position, barefoot, with their knees completely extended and the soles of their feet in contact with the sit-and-reach box roughly 15.2 cm apart. Each firefighter was asked to inhale and, when exhaling, to drop the head between the arms and slowly reach as far forward as possible, holding the stretched position for approximately two seconds. Firefighters were given three attempts; the most distant point reached with the fingertips was recorded.

### 2.5. Cardiovascular Health Parameters

In this current study, CVH was used as an umbrella term and was investigated using several approaches. These approaches included three main subcomponents, namely, CVD risk factors, CVH metrics, and HRV. The subcomponents of CVD risk factors and CVH metrics included some overlapping variables. The CVD risk factors and CVH metrics included age, body mass index (BMI), waist circumference (WC), body fat percentage (BF%), systolic blood pressure (SBP), diastolic blood pressure (DBP), total cholesterol (TC), high-density lipoprotein cholesterol (HDL-C), low-density lipoprotein cholesterol (LDL-C), triglycerides, non-fasting blood glucose (NFBG), physical activity, and diet. 

#### 2.5.1. Cardiovascular Health Metrics

The American Heart Association (AHA) used these seven CVH metrics to classify individuals as having a good index for cardiovascular health or a poor index. The CVHI was inversely related to all-cause mortality and cardiovascular events [41]. For this study, CVHI was classified as “poor” if two or fewer CVH metrics were classified as ideal and classified as “good” if firefighters had five to seven metrics rated ideal [41]. Firefighters with 3 to 4 metrics that were classified as ideal were categorized as having an intermediate CVHI. The metrics used for the CVHI included BMI, blood pressure, TC, NFBG, physical activity, cigarette smoking and diet. These factors had the same cut-off values as the CVD risk factors previously described [42,43]. 

Good BMI was classified as a BMI ≤ 24.9 kg·m^−2^, intermediate if BMI was between 25.0 and 29.9 kg·m^−2^ (overweight) and poor if ≥30 kg·m^−2^ (obese) [39,43,44]. Obesity was further classified as class I if BMI was between 30 kg·m^−2^ and 34.9 kg·m^−2^, class II if between 35 kg·m^−2^ and 39.9 kg·m^−2^ and class III if above 40 kg·m^−2^. Ideal blood pressure was classified as SBP ≤ 120 mmHg and DBP ≤ 90 mmHg, intermediate health (prehypertension) as an SBP between 121 and 139 mmHg and/or DBP between 81 and 89 mmHg, or controlled through hypertensive medication, and hypertensive as an SBP ≥ 140 mmHg and/or a DBP ≥ 90 mmHg, respectively [39,41,42,43]. Hypertension was further classified as stage 1 if SBP was between 140 mmHg and 159 mmHg and DBP between 90 and 99 mmHg, stage 2 if SBP ranged between 160 and 179 mmHg and DBP ranged between 100 mmHg and 109 mmHg and stage 3 if SBP was above 180 mmHg and DBP was above 110 mmHg [45]. Good TC was classified as a total cholesterol concentration <5.18 mmol·L, intermediate as a total cholesterol between ≥5.18 mmol·L and 6.19 mmol·L (borderline high) or controlled through medication, and poor (high) if cholesterol is above 6.2 mmol·L [39,41]. As a risk factor dyslipidaemia was considered if TC was ≥5.18 mmol·L or if on lipid-lowering medication [39]. Good NFBG concentration of <7.77 mmol·L, intermediate (prediabetic) if blood glucose concentration falls between 7.78 and 11.09 mmol·L, and poor (diabetic) if blood glucose is above 11.1 mmol·L [39,41]. Good status for cigarette smoking was classified as those that were never smokers or those who have quit for more than six months, intermediate as those who quit within six months and poor as those who are current smokers [39,41,42]. Cigarette smoking was further classified as light smokers if firefighters smoked ≤5 cigarettes a day, moderate smokers if 6 to 19 cigarettes a day and heavy smokers if ≥20 cigarettes a day [46]. Good physical activity was classified as firefighters who exercised for at least three days a week at a moderate intensity accumulating up to 150 minutes or 75 minutes of vigorous-intensity physical activity, intermediately active if they exercised for 1–149 minutes a week and physically inactive for those who do not exercise at all during a week [41,42,43]. Diet encompassed five components, namely, fruit and vegetable intake, fish intake, fibre-rich whole grains, sodium intake and sugar-sweetened-beverage intake. Diet was scored as good if four or five components were good, intermediate 2–3 components were good and poor if 0–1 components were good [41,43].

#### 2.5.2. Cardiovascular Disease Risk Score

Framingham risk [47] and lifetime and 10-year atherosclerotic cardiovascular disease (ASCVD) were calculated to assess the cardiovascular risk of firefighters [44,48]. 

#### 2.5.3. Heart Rate Variability

Heart rate variability (HRV) was measured at rest using the Polar™ (Polar Electro Oy, Kempele, Finland) H10 heart rate monitor. The HRV data were analyzed using the Kubio© Software version 3.4.3, where the results were then exported and captured onto the data collection sheet. The measures used were the variability of N-N intervals (HRV), the standard deviation of all normal-to-normal (NN) intervals (SDNN), root-mean-square of successive differences (RMSSD), low-frequency (LF), high frequency (HF) ranges and the ratio (LF/HF) [49,50]. 

### 2.6. Statistical Analysis

The data were analysed using SPSS^®^ software, version 28 (Chicago, IL, USA). The data were collected, coded, and cleaned for errors using the double-entry method on Microsoft Excel (version 16, 2019). Shapiro–Wilks test indicated that the physical fitness variables were normally distributed and that the majority of CVH metrics and CVD risk factors were not normally distributed. Descriptive statistical analyses, such as the means and standard deviations, were performed for the measures of physical fitness and medial, and 25th and 75th percentiles were used for CVH parameters. Independent samples T-tests and analysis of variance (ANOVA) were conducted to determine differences between physical fitness parameters based on sex and CVHIs. Univariable and multivariable linear regressions were conducted on the continuous data measures of physical fitness and CVH, and multinomial regression was conducted on CVHI and physical fitness. The linear regression models were cross-validated using the holdout method, randomly dividing the data into an 80% training and 20% test split. Model prediction quality was presented as R^2^ difference between the split. For the multivariable models, the hierarchical methods were preferred; covariates adjusted for in model 2 were age and sex, and in model 3, weekly physical activity and height were added to the model. Root-mean-square error (RMSE) was used to assess the model quality of each analysis conducted. The following equation was used to calculate RMSE:

$$\mathrm{RMSE}=\frac{\sqrt{\sum {\left(P-O\right)}^{2}}}{n-2}$$

For the regression analysis, steps were taken to ensure that there was no multicollinearity present in the results. Firstly, collinearity was assessed using correlations to ensure autocorrelation was not present and deemed acceptable if all values had correlation coefficients less than 0.8. Lastly, the variance inflation factor (VIF) was calculated for each variable to ensure multicollinearity was not present with the subsequent entry of variables in each model. A VIF of less than 5 was considered acceptable. Linear least absolute shrinkage and selection operator (LASSO) regression was also used to build a prediction model for each CVH parameter to reduce the number of predictors. To ensure cross-validation of the model and evaluate the predictive ability of the model a five-fold cross-validation method was used. For reporting, the more parsimonious model within 1 standard error of the optimal model was preferred. Indicators (physical fitness) with non-zero coefficients were reported only. A *p*-value of <0.05 was used to indicate statistical significance. 

## 3. Results

Table 1 presents the descriptive statistics and prevalence of CVD factors and CVHI in firefighters according to sex and age group. In total, 11.4% of firefighters reported having a good CVHI, 55.0% had an intermediate CVHI, and 34.0% had a poor CVHI. More males had a poor CVHI than females, especially those in the oldest age groups. The median age of the firefighters was 38.0 (38.0 and 48.0) years. Female firefighters had a higher BMI and BF%, which was seen in the older groups of firefighters as well. Firefighters’ cardiovascular health decreased as they aged, especially male firefighters. 

Table 2 indicates the mean physical fitness levels and differences between sex in firefighters. Male firefighters were stronger, leaner, and had more muscular stamina compared to female firefighters. Female firefighters had higher cardiorespiratory fitness than male firefighters.

Table 3 indicates the mean physical fitness levels and differences between CVHI in firefighters. Firefighters with a good CVHI had a higher cardiorespiratory fitness level, push-up and sit-up capacity, and sit-and-reach score but had the lowest grip and leg strength. Surprisingly, firefighters with poor CVHI had the highest LBM.

Table 4 describes the association between physical fitness and CVH in firefighters. AbV̇O_2max_ was significantly associated with systolic blood pressure (R^2^ = 2.5%), NFBG (R^2^ = 2.7%), HDL-C (R^2^ = 4.0%), Framingham risk score (R^2^ = 4.1%), and weekly MET minutes (R^2^ = 2.9%). After adjustment for age, sex, weekly METs, and height, every one standard deviation increase in abV̇O_2max_ was associated with a decrease of 0.249 and 0.268 standard deviations for SBP and HDL-C. RelV̇O_2max_ was significantly associated with SBP (R^2^ = 12.5%), DBP (R^2^ = 15.0%), NFBG (R^2^ = 8.9%), TC (R^2^ = 6.9%), LDL-C (R^2^ = 4.2%), HDL-C (R^2^ = 5.4%), triglycerides (R^2^ = 17.5%), and Framingham risk score (R^2^ = 29.7%). In model 3, every one standard deviation increase in relV̇O_2max_ was associated with a decrease of 0.256, 0.391, 0.290, 0.290, 0.427 and 0.117 standard deviations in SBP, DBP, NFBG, HDL-C, triglycerides and Framingham risk score, respectively. Based on muscular strength and endurance, after adjustment for age, sex, weekly METs, and height, an increase of one standard deviation in leg strength was associated with an increase in SBP, triglycerides, and weekly METs by 0.186, 0.192, and 0.226 standard deviations, respectively, and a decrease of 0.188 standard deviation in HDL-C concentration. One standard deviation increase in push-ups and sit-up capacity was associated with a decrease of 0.138 and 0.176 standard deviations for DBP and a decrease of 0.179 and 0.241 standard deviations in triglyceride concentration, respectively. 

Based on CVH, age was significantly associated with abV̇O_2max_ (R^2^ = 11.4%), relV̇O_2max_ (R^2^ = 30.5%), leg strength (R^2^ = 4.9%), sit-ups (R^2^ = 23.2%), push-ups (R^2^ = 20.2%), sit-and-reach (R^2^ = 4.0%), and LBM (R^2^ = 2.4%). After adjustment for sex, weekly METs and height, every one standard deviation in age decreased abV̇O_2max_, relV̇O_2max_, leg strength, push-ups, sit-ups, sit-and-reach, and LBM by 0.316, 0.552, 0.196, 0.451, 0.451, 0.199, and 0.178 standard deviations, respectively. Body mass index was significantly associated with abV̇O_2max_ (R^2^ = 19.1%), relV̇O_2max_ (R^2^ = 61.2%), leg strength (R^2^ = 1.7%), push-ups (R^2^ = 8.6%), sit-ups (R^2^ = 14.0%), sit-and-reach (R^2^ = 6.2%), and LBM (R^2^ = 10.6%). After adjustment for covariates, for every one standard deviation increases in BMI, abV̇O_2max_, relV̇O_2max_, push-ups, sit-ups, and sit-and-reach decreased by 0.730, 0.153, 0.252, and 0.233 standard deviations, respectively. Bodyfat percentage was significantly associated with abV̇O_2max_ (R^2^ = 4.4%), relV̇O_2max_ (R^2^ = 24.7%), grip strength (R^2^ = 2.9%), leg strength (R^2^ = 2.7%), sit-ups (R^2^ = 10.5%), push-ups (R^2^ = 16.1%), sit-and-reach (R^2^ = 2.1%), and LBM (R^2^ = 2.8%). After adjustment for age, sex, weekly METs, and height, every one standard deviation increases in BF% decreased relV̇O_2max_, push-ups, sit-ups, and sit-and-reach by 0.631, 0.228, 0.341, and 0.240 standard deviations, respectively. 

Table 5 describes the association between physical fitness and CVHI in firefighters. Univariable analysis indicated that a one-unit increase in relV̇O_2max_, push-ups, sit-ups, and sit-and-reach increased the odds of firefighters reporting intermediate CVHI rather than a poor CVHI by 1.14 (1.09, 1.20), 1.04 (1.02, 1.06), 1.05 (1.03, 1.08), and 1.04 (1.01, 1.07), respectively. One-unit increase in relV̇O_2max_, push-ups, sit-ups, and sit-and-reach increased the odds of firefighters having a good CVHI rather than a poor CVHI by 1.41 (1.29, 1.55), 1.05 (1.02, 1.08), 1.08 (1.03, 1.12), and 1.06 (1.01, 1.11), respectively. In Model 2, after adjustment for age and sex, a one-unit increase in relV̇O_2max_, push-ups, sit-ups, and sit-and-reach increased the odds of firefighters having an intermediate CVHI rather than a poor CVHI by a factor of 1.14 (1.08, 1.21), 1.03 (1.01, 1.05), 1.04 (1.01, 1.07), and 1.04 (1.01, 1.07), respectively. In addition, a one-unit increase in abV̇O_2max_, relV̇O_2max_, and sit-ups increased the odds of firefighters reporting a good CVHI by 0.09 (0.02, 0.51), 1.38 (1.24, 1.53), and 1.06 (1.01, 1.11). In Model 3, after weekly METs and years of experience were added to the model, a one-unit increase in relV̇O_2max_, sit-ups, and sit-and-reach increased the odds of firefighters reporting intermediate health rather than poor CVHI by 1.19 (1.11, 1.28), 1.04 (1.01, 1.07), and 1.04 (1.01, 1.07), respectively. A one-unit increase in relV̇O_2max_ and sit-ups increased the odds of firefighters reporting a good CVHI by 1.49 (1.33, 1.69) and 1.06 (1.01, 1.11), respectively.

Table 6 shows the relationship between measures of physical fitness and HRV in firefighters. Univariate regression found that abV̇O_2max_ and relV̇O_2max_ explained 26.8% and 9.3%, 27.3% and 22.8%, 25.9% and 22.5%, and 3.7% and 7.6% of the variation in HRV, SDNN, RMSSD, and LF/HF ratio, respectively. After adjustment for age, sex, weekly METs, and height, one standard deviation increases in abV̇O_2max_ and relV̇O_2max_ increased in HRV, SDNN, and RMSSD by 0.608 and 0.565, 0.478 and 0.522, and 0.516 and 0.559 standard deviations, respectively, and decreased LF/HF ratio by 0.254 and 0.303 standard deviations. Push-ups and sit-ups explained 8.4% and 6.3% of the variation in SDNN, and after adjustment for age, sex, weekly METs, and height, one standard deviation increase in push-up and sit-up capacity increased SDNN by 0.161 and 0.129 standard deviations, respectively.

Table 7 describes the LASSO results for key indicators of physical fitness associated with CVH in firefighters. The results of the LASSO regression reported that relV̇O_2max_ and LBM were the most significant indicators of SBP in firefighters, with the highest predicted model accuracy (0.905 vs. 0.923). RelV̇O_2max_ and sit-ups were significant indicators of DBP in firefighters, with the highest predicted model accuracy (0.903 vs. 0.930). AbV̇O_2max_ and relV̇O_2max_ were the most significant indicators of NFBG with the highest predicted model accuracy (0.954 vs. 0.969). RelV̇O_2max_ was the most significant indicator of TC and LDL-C concentrations; however, model predictive accuracy was low for TC (0.965 vs. 0.602) and high for LDL-C (0.989 vs. 0.996). For HDL-C abV̇O_2max_, relV̇O_2max_, leg strength, and push-up capacity were the most significant indicators, with the highest predicted model accuracy (0.894 vs. 0.930). RelV̇O_2max_ was the most significant indicator of triglycerides in firefighters, with the highest predicted model accuracy (0.871 vs. 0.901). Leg strength was the most significant indicator of MET minutes, with the highest predicted model accuracy (0.993 vs. 0.999). For CVHI, abV̇O_2max_ was the most significant indicator of a good CVHI, with the highest predicted model accuracy (0.942 vs. 0.958). AbV̇O_2max_, relV̇O_2max_, and leg strength were the most significant indicators of HRV in firefighters; however, model predictive accuracy was low (0.635 vs. 0.649). AbV̇O_2max_ and relV̇O_2max_ were the most significant indicators for SDNN and RMSSD; however, the model predictive accuracy was low for both SDNN (0.476 vs. 0.479) and RMSSD (0.518 vs. 0.527). Leg strength was the most significant indicator of HF ratio in firefighters, with the highest predicted model accuracy (0.995 vs. 0.999). AbV̇O_2max_ and relV̇O_2max_ were the most significant indicators for LF/HF ratio, with the highest predicted model accuracy (0.911 vs. 0.943).

## 4. Discussion

The results of this present study indicated that higher cardiorespiratory fitness, muscular strength and endurance, and body composition were associated with CVH status, particularly, blood pressure, and blood lipid concentrations in firefighters. In addition, age and obesity were inversely related to all measures of physical fitness in firefighters. Firefighters with higher cardiorespiratory fitness had a better HRV. Moreover, after LASSO regression was performed, relative cardiorespiratory fitness was the most significant indicator of better overall CVH in firefighters. The results support earlier research that found a relationship between physical fitness and CVH [30,31,32]. Moreover, physical fitness and CVH were modestly associated, where a decrease in one, may, present as a decline in the other. This becomes more prominent as firefighters age, which has been associated with a reduction in both physical fitness and CVH in firefighters [30,31,32]. The results are consistent with previous studies that indicated that aged and obese firefighters are more likely to have poor levels of physical fitness, and firefighters with higher levels of physical fitness had more favourable CVH parameters, such as body composition, blood pressure, blood lipid, and blood glucose concentrations [30,31,32,33].

The current results found that physical fitness was inversely associated with cholesterol concentrations and blood pressure. This is supported by previous studies, where it had been reported that HDL-C, SBP, and DBP were significantly associated with most measures of physical fitness [51,52,53,54,55]. The opposite holds true as well, as improvements in physical fitness would improve overall cardiovascular health, which has been shown in previous studies [7,30,31,32]. This relationship between physical fitness and CVH may be bidirectional and suggests that physical fitness influences CVH and, in turn, CVH influences physical fitness [51,52,55]. This is seen in the present results, where, collectively, increased adiposity and blood pressure and worsened blood cholesterol concentrations were most likely to be attributed to lower levels of physical fitness in firefighters. These observations are supported by a recently published systematic review, which indicated that adiposity, blood pressure, and cholesterol concentrations were bi-directionally associated with physical fitness [56].

The results indicated that relVO_2max_ and abVO_2max_ were significantly and negatively associated with blood pressure, NFBG, lipid profile, Framingham risk score, and CVHI. In addition, LASSO results indicated that relVO_2max_ was consistently a key indicator of good CVH parameters in firefighters. This is consistent with previous studies, which showed that firefighters with higher cardiorespiratory fitness levels had better overall cardiovascular health compared to less fit firefighters [7,30,31]. Regular physical activity is a prerequisite to improving cardiorespiratory fitness. Moreover, DeFina et al. [57] reported that cardiorespiratory fitness was a reliable marker of cardiovascular health and functioning, indicating good oxygen transport and absorption. This suggests that firefighters with favourable cardiorespiratory fitness had better cardiovascular functioning, which is shown by a favourable CVHI and risk profile. The added benefits of regular physical activity would be the positive effect exercise has on blood pressure, blood glucose and lipid concentrations, especially associated with reductions in LDL-C and increases in HDL-C [30,31,34]. 

We found that grip strength was significantly and negatively associated with HDL-C. Leg strength was significantly associated with SBP, HDL-C, triglycerides, and weekly MET minutes. After adjustment for covariates, leg strength remained significantly associated with SBP, HDL-C, and MET minute. In addition, leg strength remained a significant indicator for HDL-C and weekly MET minutes. Gubelmann et al. [54] noted that grip strength was significantly correlated with blood pressure, LDL-C, triglycerides, and blood glucose. Although grip strength was only associated with HDL-C in this current study, leg strength reported similar associations as Gubelmann et al. [54]. Similarly, Yamada et al. [58] reported that grip strength was significantly correlated with HDL-C. Carter et al. [59] found that resistance training reduced arterial blood pressure in healthy adults. This was supported by a study that reported in hypertensive older adults, strength training normalised their blood pressure [60]. Singh et al. [61] reported that leg strength was associated with cardiovascular disease mortality. This suggests that maintaining strength levels in adults may have a positive effect on all cardiovascular parameters, which is particularly important for firefighters, given the physically strenuous nature of their occupations [1]. Studies indicated that firefighters that participated in regular physical activity were significantly stronger than those who were sedentary or participated in lower levels of physical activity [2,62,63]. 

Push-ups and sit-ups were significantly and inversely associated with SBP, DBP, NFBG, TC, triglycerides, Framingham risk score, and CVHI and positively associated with weekly MET minutes. Moreover, in the LASSO analysis, push-ups remained a key indicator of HDL-C, and sit-ups remained a key indicator of DBP. Lin et al. [53] reported that push-up capacity was significantly associated with average variability in SBP, but push-ups were not significantly associated with any measures of blood pressure in military personnel. This is supported by Vaara et al. [51], who reported that muscular endurance was associated with triglycerides, LDL-C, glucose, blood pressure, and HDL-C. Similarly, Shina and Ha [52] reported that sit-ups were significantly correlated to SBP and DBP. Yang et al. [55] reported that push-up capacity was significantly related to future cardiovascular events in male firefighters, supporting that firefighters that focused on increasing their muscular stamina may have an improved CVH. Firefighters that have higher levels of muscular endurance are likely, to participate in regular physical activity that is of moderate intensity [16,34], contributing to higher muscular endurance and better cardiovascular health status. Nonetheless, higher muscular endurance seems to be a good marker of better cardiovascular health in firefighters.

We found that age and obesity had significant linear and negative associations of all measures of physical fitness, except grip strength, and remained significant after adjustment of covariates. This was reported to be similar among previous studies, where it was consistently reported that all measures of physical fitness decreased as firefighters aged and fat mass increased [24,25,29,64]. Moreover, overeating while on duty and physical inactivity may compound this issue, as physical fitness is essential in maintaining body composition, musculoskeletal health, muscular strength and endurance, especially as firefighters age [16,34,65]. Ageing has been associated with a progressive decrease in muscle mass and bone mineral density, which has a negative effect on physical fitness in firefighters [25,29,66]. This is supported by studies which reported muscular endurance and force production decreased as firefighters aged [25,67], explaining why, in this current study, aged firefighters performed significantly worse on all physical fitness tests. Furthermore, a progressive decrease in vascular elasticity increases blood pressure, therefore, the afterload that the heart is required to overcome, ultimately negatively affecting cardiac output and reducing cardiovascular fitness [68,69,70]. Obesity compounds this for all aspects of physical fitness, as it increases the non-functional mass firefighters are required to carry [70,71]. This places an additional strain on the cardiorespiratory system, reducing cardiac output [72]. Similarly, increased fat mass increases the amount of weight firefighters are required to move during bodyweight movements, such as push-ups and sit-ups [25,73]. This was seen in the tests for lower body strength as well, where firefighters that had a higher BMI and BF% had a lower level of leg strength. Obese firefighters, or those with higher fat mass, force them to overcome more non-functional mass, reducing their absolute leg strength [25,64]. 

The results showed that HRV, SDNN, RMSSD, and LF/HF ratio were significantly associated with cardiorespiratory fitness, muscular strength, and endurance in firefighters. In addition, leg strength, push-ups, and sit-ups were significant indicators in the LASSO regression of HRV, LF, and HF in firefighters. Porto et al. [74] found that RMSSD and LF/HF ratio were significantly related to cardiorespiratory fitness in firefighters. In contrast, Lesniak et al. [75] found that there were no measures of HRV that were significantly related to cardiorespiratory fitness in firefighters. However, the study noted that LF, HF, and LF/HF ratio were significantly related to deadlift strength in firefighters. Grant et al. [76] reported that HRV, RMSSD, and LF significantly improved after military personnel engaged in an exercise intervention. Engaging in regular physical activity may promote parasympathetic dominance and decrease sympathetic cardiac control, significantly improving cardiac functioning in firefighters [50,74,76]. Firefighters who have higher cardiorespiratory fitness may have a lower heart rate, thus, higher variability in their normal-to-normal intervals, increasing their HRV metrics [50,75,77]. Moreover, firefighters with better HRV indices are likely fitter, stronger, and less stressed than those with worse HRV indices [50,74,77]. These findings highlight the potential use of measures such as HRV to assess the CVH, cardiorespiratory functioning, and stress states of firefighters; however, more studies are needed in this area.

### Strengths and Limitations

This was the first study investigating the association between physical fitness and cardiovascular health in firefighters in the CoCTFRS. The measures for physical fitness and cardiovascular health were objectively measured by trained researchers using standardised and validated instruments [34]. Furthermore, this paper adds novel information on physical fitness and CVH in firefighters in an area that is understudied, particularly in a South African context. There are, however, several limitations of this present study. First, the cross-sectional study design limits our ability to infer causal relationships. Secondly, female firefighters were under-represented, limiting the generalizability of our results to the female firefighter population. Lastly, cardiorespiratory fitness was measured using a non-exercise estimation, not using lab or field testing.

## 5. Conclusions

This present study provides evidence that multiple components of physical fitness and cardiovascular health are significantly associated with this population of firefighters. Overall, cardiorespiratory fitness, muscular strength, and endurance were significantly related to a better overall cardiovascular health profile. However, cardiorespiratory fitness may be the key physical fitness parameter to ensure good CVH in firefighters. Moreover, aged and obese firefighters have the poorest overall level of physical fitness and CVH. Ageing, accumulated body fat, and physical inactivity likely serve as catalysts that lead to an increase in body fat and a decline in physical fitness and, subsequently, a reduction in CVH. This study adds novel research results into a scarcely studied research area, especially in a South African context and highlights the importance of physical fitness in maintaining cardiovascular health and vice versa. In particular, the impact of ageing and obesity on physical fitness and all measures of physical fitness on CVH among firefighters is highlighted. Implementing regular physical activity, with a combination of aerobic and resistance training, may improve and maintain the cardiovascular health and well-being of firefighters, which is increasingly important as firefighters age.

### Recommendations

Intervention studies are warranted to examine the effect of maintenance or the improvement of key physical fitness parameters on cardiovascular health. A more representative sample of female firefighters should be included to assess the validity and generalizability of study findings to the entire population of firefighters in the CoCTFRS. In addition, a larger sample to increase the strength of the analysis and model prediction accuracy is warranted.

## Figures and Tables

**Table 1:** Descriptive characteristics, prevalence of cardiovascular disease risk factors, and cardiovascular health metrics in firefighters according to sex and age group.

	Good CVHI		Intermediate Health		Poor CVHI	
	N	%	N	%	N	%
Total Firefighters (n = 309)	34	11.0	170	55.0	105	34.0
Male (n = 275)	24	56.0	154	56.0	97	35.3
Female (n = 34)	10	29.4	16	47.1	8	23.5
20–29 years (n = 72)	16	22.2	41	56.9	15	20.8
30–39 years (n = 95)	11	11.6	56	58.9	28	29.5
40–49 years (n = 83)	6	7.2	45	54.2	32	38.6
50–65 years (n = 58)	1	1.7	27	46.6	30	51.7
Cardiovascular health metric/cardiovascular disease risk factor	N	*X̃* (p25th–p75th)			N	%
Age ^¶ §^	309	38.0 (38.0, 48.0)			93	30.1
Body mass index	309	27.1 (24.1, 30.4)				
Obese ^¶^ ***	-	-			86	27.8
*Ideal* *	-	-			94	30.4
*Overweight* **	-	-			129	41.8
*Obese class I*	-	-			67	21.7
*Obese class II*	-	-			14	4.5
*Obese class III*	-	-			4	1.3
High body fat percentage ^¶^	309	20.2 (14.9, 27.2)			84	27.2
Central obesity ^¶^	309	93.0 (84.3, 101.0)			159	51.5
Hypertension ^¶^ ***	-	-			144	46.6
*Ideal* *	-	-			35	11.3
*Pre-hypertensive* **	-	-			130	42.1
*Hypertension stage 1*	-	-			114	36.9
*Hypertension stage 2*	-	-			13	4.2
*Hypertension stage 3*	-	-			4	1.3
Systolic hypertension	309	137.3 (125.0, 145.5)			133	43.0
Diastolic hypertension	309	81.7 (74.2, 90.8)			86	27.8
Dyslipidaemia ^¶^					109	35.3
Total cholesterol	309	4.6 (3.9, 3.4)				
*Ideal (<5.18 mmol·L)* *	-	-			211	68.3
*Borderline high (5.18–6.19 mmol·L)* **	-	-			69	22.3
*High (>6.2 mmol·L)* ***	-	-			29	9.4
Low-density lipoprotein cholesterol ^¶^	309	2.6 (2.1, 3.4)				
*Ideal (<3.35 mmol*·*L)*	-	2.3 (1.9, 2.8)			228	73.8
*Borderline high (3.35–4.12 mmol*·*L)*	-	2.4 (2.4, 3.3)			52	16.8
*High (4.13–4.9 mmol*·*L)*	-	1.2 (1.1, 1.5)			16	5.2
*Very high (>4.9 mmol*·*L)*	-	3.2 (2.3, 3.7)			12	3.9
High-density lipoprotein cholesterol	309	1.2 (1.0, 1.4)			65	21.0
*Low (<1.03 mmol·L)*	-	1.2 (1.1, 1.4)			70	22.7
*Desirable (1.3–1.55 mmol·L)*	-	1.2 (0.8, 1.9)			184	59.5
*High (>1.55 mmol·L)*	-	1.2 (1.1, 1.5)			55	17.8
Hypertriglyceridemia ^¶^	309	1.4 (0.9, 2.2)			119	38.5
*Normal (<1.7 mmol·L)*	-	1.1 (0.8, 1.7)			191	61.8
*Borderline high (1.7–2.25 mmol·L)*	-	1.2 (0.8, 1,9)			52	16.8
*High (2.26–5.64 mmol·L)*	-	1.6 (1.1, 2.3)			60	19.4
*Very high (>5.65 mmol·L)*	-	2.0 (1.3, 2.8)			6	1.9
Non-fasting blood glucose	309	5.4 (4.9, 6.2)				
*Normal (<7.77 mmol·L)* *	-	5.2 (4.6, 5.9)			289	93.5
*Prediabetes (7.8–11.1 mmol·L)* **	-	5.4 (4.8, 6.0)			15	4.9
*Diabetic (>11.1 mmol·L)* ***	-	5.4 (4.9, 6.3)			5	1.6
Diet	309	10.0 (8.0, 11.0)			-	-
*Ideal diet* *	-	-			2	0.6
*Intermediate diet* **	-	-			55	17.6
*Poor diet* ***	-	-			252	78.3
Weekly physical activity (METs) ^¶^	309	2240.0 (1165.0, 3986.5)			179	57.9
*Physically active* *	-	-			96	42.1
*Insufficiently active* **	-	-			83	26.9
*Completely inactive* ***	-	-			96	31.1
Cigarette smoking ^¶^ ***	22	10.0 (5.0, 20.0)			108	35.0
*Never* *	-	-			201	65.0
*Quit within 6 months* **	-	-			1	0.9
*Light smoker (<5)*	-	-			27	25.0
*Moderate smoker (≥6–19)*	-	-			46	42.6
*Heavy smoker (>20)*	-	-			30	27.8
Framingham risk score	309	1.1 (0.2, 5.9)			-	-
*Low (<10%)*	-	-			278	86.3
*Moderate (10–19%)*	-	-			23	7.1
*High (≥20)*	-	-			8	2.5
Lifetime ASCVD risk score ^▲^	304	50.0 (39.0, 50.0)			-	-
10-year ASCVD risk score ^▼^	138	5.5 (2.4, 9.9)			-	-
*Low risk (<5%)*	-	-			62	19.3
*Borderline risk (5–7.5%)*	-	-			26	8.1
*Intermediate risk (7.5–20%)*	-	-			45	14.0
*High risk (≥20%)*	-	-			3	0.9

Note: *X̃*—median; p25th–p75th–25th percentile to 75th percentile; CVHI—cardiovascular health index; ^¶^—indicates positive cardiovascular disease risk factor; *—indicates good cardiovascular health metric; **—indicates intermediate health metric; ***—indicates poor cardiovascular health metric; ^§^—indicates males ≥ 45 years and females ≥ 55 years; ^▲^—indicates 5 firefighters were not included in lifetime ASCVD risk score due to being over 60 years old. ^▼^—indicates that 171 firefighters were removed due to being under the age of 40 years.

**Table 2:** Descriptive characteristics of firefighters’ physical fitness according to sex.

			Sex				
	Total Firefighters		Males		Females		
Physical fitness	N	x̄ ± SD	N	x̄ ± SD	N	x̄ ± SD	*p* ^§^
abV̇O_2max_ (L·min)	309	3.4 ± 0.3	275	3.4 ± 0.3	34	3.3 ± 0.3	**0.014**
relV̇O_2max_ (mL·kg·min)	309	42.3 ± 6.1	275	41.9 ± 5.9	34	45.1 ± 7.4	**0.005**
Grip strength (kg)	304	89.7 ± 17.5	270	92.9 ± 15.4	34	64.6 ± 12.0	**<0.001**
Right grip strength (kg)	304	45.2 ± 9.2	270	46.7 ± 8.2	34	32.7 ± 6.3	**<0.001**
Left grip strength (kg)	304	44.5 ± 8.9	270	46.1 ± 7.9	34	31.9 ± 6.1	**<0.001**
Leg strength (kg)	304	116.3 ± 29.3	270	120.6 ± 26.9	34	82.0 ± 23.9	**<0.001**
Push-ups (rpm)	304	30.9 ± 13.9	270	31.4 ± 14.3	34	26.7 ± 10.1	0.105
Sit-ups (rpm)	304	28.4 ± 10.4	270	28.7 ± 10.4	34	25.9 ± 9.9	0.140
Sit-and-reach (cm)	304	42.8 ± 9.1	270	42.3 ± 9.1	34	46.9 ± 8.7	**0.006**
Lean body Mass (kg)	309	60.4 ± 9.8	275	62.4 ± 8.1	34	43.8 ± 5.7	**<0.001**

Note: Bold indicates statistical significance. V̇O_2max—_oxygen consumption; L·min—litres per min; mL·kg·min—millilitres per kilogram per minute; kg—kilogram; cm—centimetres; rpm—repetitions per minute. ^§^—indicates independent samples *t*-test that was conducted.

**Table 3:** Descriptive characteristics of firefighters’ physical fitness according to cardiovascular health index.

	Cardiovascular Health Index						
	Poor CVHI		Intermediate CVHI		Good CVHI		
Physical fitness	N	x̄ ± SD	N	x̄ ± SD	N	x̄ ± SD	*p* ^#^
abV̇O_2max_ (L·min)	101	3.4 ± 0.3	174	3.4 ± 0.3	34	3.3 ± 0.2	0.253
relV̇O_2max_ (mL·kg·min)	101	39.0 ± 4.7	174	42.9 ± 5.8	34	49.3 ± 4.9	**<0.001**
Grip strength (kg)	99	89.7 ± 15.9	174	91.4 ± 17.6	34	80.9 ± 19.2	**0.006**
Right grip strength (kg)	99	45.2 ± 8.6	171	45.9 ± 9.1	34	41.3 ± 10.2	**0.026**
Left grip strength (kg)	99	44.6 ± 8.1	171	45.5 ± 9.0	34	39.7 ± 9.5	**0.002**
Leg strength (kg)	99	118.1 ± 26.5	171	116.2 ± 29.6	34	111.7 ± 31.4	0.538
Push-ups (rpm)	99	26.4 ± 13.2	171	32.7 ± 13.8	34	34.6 ± 12.1	**<0.001**
Sit-ups (rpm)	99	24.5 ± 11.0	171	29.9 ± 9.9	34	32.1 ± 5.8	**<0.001**
Sit-and-reach (cm)	99	40.3 ± 9.3	171	43.9 ± 8.9	34	45.0 ± 8.9	**0.003**
Lean body Mass (kg)	100	62.5 ± 9.1	174	60.5 ± 9.8	34	53.3 ± 7.5	**<0.001**

Note: Bold indicates statistical significance. V̇O_2max_—oxygen consumption; L·min—litres per min; mL·kg·min—millilitres per kilogram per minute; kg—kilogram; cm—centimetres; rpm—repetitions per minute; ^#^—indicates the analysis of variance conducted.

**Table 4:** Linear regression indicating the association between physical fitness and cardiovascular health in firefighters.

	Univariate Linear Models ^a^							Multivariate Linear Models ^b^											
	Model 1							Model 2 ^c^						Model 3 ^d^					
Variables	B	SE	β	R^2^	RMSE	CV ^▲^	*p*	B	SE	β	R^2^	RMSE	*p*	B	SE	β	R^2^	RMSE	*p*-Value
Exploratory variable: abV̇O_2max_																			
Systolic blood pressure (mmHg)	8.738	3.106	0.159	0.025	15.254	1.3	**0.005**	14.101	3.111	0.247	0.162	14.189	**<0.001**	13.684	3.355	0.249	0.164	14.210	**<0.001**
Diastolic blood pressure (mmHg)	−0.754	2.373	0.096	0.000	11.656	0.0	0.751	-	-	-	-	-	-	-	-		-	-	-
Non-fasting blood glucose (mmol·L)	−0.808	0.277	−0.164	0.027	1.360	10.9	**0.004**	−0.511	0.293	−0.095	0.064	1.338	0.082	-	-	-	-	-	-
Total cholesterol (mmol·L)	−0.320	0.259	−0.070	0.005	1.274	0.8	0.218	-	-	-	-	-	-	-	-	-	-	-	-
Low-density lipoprotein cholesterol (mmol·L)	−0.322	0.217	−0.081	0.007	1.066	0.09	0.139	-	-	-	-	-	-	-	-	-	-	-	-
High-density lipoprotein (mmol·L)	−0.276	0.077	−0.201	0.040	0.377	11.0	**<0.001**	−0.283	0.080	−0.193	0.115	0.363	**<0.001**	−0.365	0.085	−0.268	0.137	0.359	**<0.001**
Triglycerides (mmol·L)	0.056	0.225	0.014	0.000	1.104	1.2	0.802	-	-	-	-	-	-	-	-	-	-	-	-
Framingham risk score	−0.011	0.003	−0.203	0.041	5.163	3.2	**<0.001**	0.005	0.005	0.091	0.663	3.064	0.322	-	-	-	-	-	-
Weekly MET minutes	1746.3	573.22	0.172	0.029	2811.269	4.6	**0.003**	1306.261	614.100	0.114	0.044	2799.691	**0.034**	1193.3	658.262	0.117	0.044	2803.237	0.071
																			
Exploratory variable: relV̇O_2max_																			
Systolic blood pressure (mmHg)	−0.888	0.170	−0.353	0.125	14.511	3.7	**<0.001**	−0.671	0.161	−0.267	0.153	14.262	**<0.001**	−0.642	0.170	−0.256	0.158	14.278	**<0.001**
Diastolic blood pressure (mmHg)	−0.735	0.100	−0.388	0.150	10.754	19.6	**<0.001**	−0.637	0.121	−0.336	0.166	10.681	**<0.001**	−0.742	0.126	−0.391	0.186	10.569	**<0.001**
Non-fasting blood glucose (mmol·L)	−0.067	0.012	−0.299	0.089	1.314	6.5	**<0.001**	−0.055	0.015	−0.246	0.090	1.316	**<0.001**	−0.065	0.016	−0.290	0.108	1.309	**<0.001**
Total cholesterol (mmol·L)	−0.054	0.011	−0.262	0.069	1.237	−11.8	**<0.001**	−0.027	0.014	−0.130	0.116	1.205	**0.048**	−0.026	0.014	−0.126	0.116	1.207	0.068
Low-density lipoprotein cholesterol (mmol·L)	−0.036	0.010	−0.206	0.042	1.049	−15.5	**<0.001**	−0.017	0.012	−0.095	0.078	1.031	0.157	-	-	-	-	-	-
High-density lipoprotein (mmol·L)	0.015	0.003	0.232	0.054	0.371	6.2	**<0.001**	0.015	0.067	0.242	0.118	0.363	**<0.001**	0.018	0.004	0.290	0.134	0.359	**<0.001**
Triglycerides (mmol·L)	−0.077	0.012	−0.422	0.175	0.999	3.8	**<0.001**	−0.064	0.011	−0.355	0.194	0.995	**<0.001**	−0.077	0.012	−0.427	0.231	0.976	**<0.001**
Framingham risk score	−0.637	0.056	−0.545	0.297	4.429	6.2	**<0.001**	−0.100	0.034	−0.117	0.671	3.027	**0.004**	−0.100	0.036	−0.117	0.674	3.018	**0.006**
Weekly MET minutes	0.000	0.000	0.172	0.010	2811.269	1.2	0.073	-	-	-	-	-	-	-	-	-	-	-	-
										-				-	-	-	-		-
Exploratory variable: Grip strength														-					
Systolic blood pressure (mmHg)	0.079	0.050	0.090	0.008	15.385	1	0.119	-	-	-	-	-	-	-	-	-	-	-	-
Diastolic blood pressure (mmHg)	−0.038	0.038	−0.057	0.003	11.499	8.1	0.322	-	-	-	-	-	-	-	-	-	-	-	-
Non-fasting blood glucose (mmol·L)	0.002	0.005	0.021	0.000	1.376	1.8	0.363	-	-	-	-	-	-	-	-	-	-	-	-
Total cholesterol (mmol·L)	−0.001	0.004	−0.015	0.000	1.282	0.0	0.798	-	-	-	-	-	-	-	-	-	-	-	-
Low-density lipoprotein cholesterol (mmol·L)	0.001	0.004	0.009	0.000	1.0469	0.1	0.881	-	-	-	-	-	-	-	-	-	-	-	-
High-density lipoprotein (mmol·L)	−0.004	0.001	−0.163	0.027	0.376	−9.6	**0.004**	−0.001	0.001	−0.034	0.081	-	0.604	-	-	-	-	-	-
Triglycerides (mmol·L)	0.002	0.004	0.028	0.001	1.107	0.1	0.633	-	-	-	-	-	-	-	-	-	-	-	-
Framingham risk score	0.012	0.017	0.039	0.002	5.279	0.0	0.501	-	-	-	-	-	-	-	-	-	-	-	-
Weekly MET minutes	6.358	9.378	0.039	0.002	2854.287	5.6	0.678	-	-	-	-	-	-	-	-	-	-	-	-
																			
Exploratory variable: Leg strength																			
Systolic blood pressure (mmHg)	0.092	0.030	0.173	0.030	15.224	1.1	**0.003**	0.101	0.033	0.189	0.127	14.448	**0.002**	0.099	0.034	0.186	0.137	14.404	**0.004**
Diastolic blood pressure (mmHg)	0.018	0.023	0.045	0.004	11.497	0.1	0.303	-	-	-	-	-	-	-	-	-	-	-	-
Non-fasting blood glucose (mmol·L)	0.001	0.003	0.028	0.001	1.375	−0.1	0.687	-	-	-	-	-	-	-	-	-	-	-	-
Total cholesterol (mmol·L)	0.000	0.003	0.005	0.000	1.282	−4.5	0.981	-	-	-	-	-	-	-	-	-	-	-	-
Low-density lipoprotein cholesterol (mmol·L)	0.000	0.002	−0.010	0.000	1.074	4.1	0.810	-	-	-	-	-	-	-	-	-	-	-	-
High-density lipoprotein (mmol·L)	−0.002	0.001	−0.219	0.048	0.373	−11.9	**<0.001**	−0.002	0.001	−0.146	0.097	0.368	**0.020**	−0.002	0.001	−0.188	0.110	0.365	**0.004**
Triglycerides (mmol·L)	0.005	0.002	0.119	0.016	1.098	−6.5	**0.038**	0.006	0.002	0.147	0.124	1.041	**0.017**	0.007	0.002	0.192	0.146	1.033	**0.003**
Framingham risk score	−0.011	0.011	−0.061	0.004	5.293	−5.2	0.291	-	-	-	-	-	-	-	-	-	-	-	-
Weekly MET minutes	22.2	5.556	0.225	0.055	2728.984	−3.6	**<0.001**	23.062	6.268	0.234	0.069	2761.658	**<0.001**	22.334	6.365	0.226	0.070	2764.178	**<0.001**
																			
Exploratory variable: Push-ups																			
Systolic blood pressure (mmHg)	−0.133	0.064	−0.119	0.014	15.368	13.7	**0.038**	−0.026	0.070	−0.024	0.100	14.677	0.708	-	-	-	-	-	-
Diastolic blood pressure (mmHg)	−0.203	0.047	−0.242	0.058	11.203	−32.1	**<0.001**	−0.109	0.053	−0.130	0.099	11.038	**0.041**	−0.116	0.053	−0.138	0.106	11.0456	**0.030**
Non-fasting blood glucose (mmol·L)	−0.004	0.006	−0.137	0.019	1.365	−10.7	**0.017**	−0.004	0.006	−0.044	0.051	1.345	0.494	-	-	-	-	-	-
Total cholesterol (mmol·L)	0.005	0.006	−0.125	0.016	1.276	−19.9	**0.029**	0.005	0.006	0.050	0.114	1.212	0.428	-	-	-	-	-	-
Low-density lipoprotein cholesterol (mmol·L)	−0.006	0.004	−0.010	0.005	1.073	12.7	0.221	-	-	-	-	-	-	-	-	-	-	-	-
High-density lipoprotein (mmol·L)	0.002	0.002	0.090	0.007	0.385	0.5	0.141	-	-	-	-	-	-	-	-	-	-	-	-
Triglycerides (mmol·L)	−0.020	0.004	−0.254	0.065	1.072	−3.2	**<0.001**	−0.014	0.005	−0.176	0.131	1.037	**0.005**	−0.014	0.005	−0.179	0.143	1.035	**0.004**
Framingham risk score	−0.157	0.020	−0.411	0.169	4.838	−7.8	**<0.001**	−0.029	0.015	−0.075	0.675	3.023	**0.049**	−0.026	0.015	−0.068	0.679	3.010	0.074
Weekly MET minutes	28.654	11.864	0.138	0.019	2824.061	2.6	**0.016**	15.876	13.571	0.077	0.031	2825.378	0.243	-	-	-	-	-	-
																			
Exploratory variable: Sit-ups																			
Systolic blood pressure (mmHg)	−0.237	0.084	−0.160	0.026	15.187	32.1	**0.005**	−0.114	0.091	−0.077	0.111	14.492	0.210	-	-		-		-
Diastolic blood pressure (mmHg)	−0.250	−0.270	−0.279	0.078	11.064	28.2	**<0.001**	−0.200	0.068	−0.179	0.116	10.899	**0.004**	−0.196	0.068	−0.176	0.122	10.913	**0.004**
Non-fasting blood glucose (mmol·L)	−0.021	0.008	−0.156	0.024	1.361	5.6	**0.007**	−0.010	0.008	−0.075	0.055	-	0.239	-	-	-	-	-	-
Total cholesterol (mmol·L)	−0.018	0.007	−0.146	0.021	1.269	8.8	**0.011**	0.001	0.008	0.006	0.101	-	0.925	-	-	-	-	-	-
Low-density lipoprotein cholesterol (mmol·L)	−0.008	0.006	−0.082	0.007	1.067	3.0	0.155	-	-	-	-	-	-	-	-	-	-	-	-
High-density lipoprotein (mmol·L)	0.003	0.002	0.074	0.006	0.385	2.2	0.198	-	-	-	-	-	-	-	-	-	-	-	-
Triglycerides (mmol·L)	−0.033	0.006	−0.305	0.093	1.056	7.6	**<0.001**	−0.026	0.006	−0.244	0.153	1.023	**<0.001**	−0.026	0.006	−0.241	0.163	1.022	**<0.001**
Framingham risk score	−0.198	0.027	−0.289	0.151	4.888	0.6	**<0.001**	−0.037	0.019	−0.073	0.674	3.028	0.051	-	-	-	-	-	-
Weekly MET minutes	02.154	15.844	0.073	0.005	2850.996	0.7	0.204	-	-	-	-	-	-	-	-	-	-	-	-
																			
Exploratory variable: Age																			
abV̇O_2max_ (L·min)	−0.009	0.001	−0.342	0.114	0.263	4.9	**<0.001**	−0.009	0.001	−0.344	0.138	0.261	**<0.001**	−0.009	0.001	−0.316	0.254	0.243	**<0.001**
relV̇O_2max_ (mL·kg·min)	−0.328	0.028	−0.552	0.305	5.125	0.3	**<0.001**	−0.327	0.028	−0.550	0.328	5.049	**<0.001**	−0.328	0.027	−0.552	0.391	4.828	**<0.001**
Grip strength (kg)	−0.139	0.098	−0.081	0.007	17.463	0.3	0.157	-	-	-	-	-	-	-	-	-	-	-	-
Right grip strength (kg)	−0.070	0.051	−0.082	0.003	9.130	0.3	**0.051**	-	-	-	-	-	-	-	-	-	-	-	-
Left grip strength (kg)	−0.069	0.050	−0.080	0.003	8.909	0.2	0.170	-	-	-	-	-	-	-	-	-	-	-	-
Leg strength (kg)	−0.621	0.158	−0.221	0.049	28.162	7.1	**<0.001**	−0.629	0.143	−0.224	0.221	25.532	**<0.001**	−0.552	0.141	−0.196	0.277	24.692	**<0.001**
Push-ups (rpm)	−0.646	0.068	−0.482	0.232	12.076	1.2	**<0.001**	−0.647	0.067	−0.483	0.242	12.021	**<0.001**	−0.616	0.070	−0.451	0.233	12.351	**<0.001**
Sit-ups (rpm)	−0.455	0.052	−0.450	0.202	9.264	10.5	**<0.001**	−0.455	0.052	−0.450	0.210	9.235	**<0.001**	−0.457	0.053	−0.451	0.211	9.273	**<0.001**
Sit-and-reach (cm)	−0.186	0.050	−0.208	0.040	8.962	4.3	**<0.001**	−0.185	0.050	−0.207	0.067	8.864	**<0.001**	−0.178	0.050	−0.199	0.080	8.825	**<0.001**
Lean body Mass (kg)	0.153	0.053	0.163	0.024	9.719	0.9	**0.007**	0.146	0.044	0.156	0.330	7.125	**<0.001**	0.167	0.037	0.178	0.541	6.614	**<0.001**
																		
Exploratory variable: body mass index																			
abV̇O_2max_ (L·min)	0.026	0.003	0.440	0.191	0.252	−8.1	**<0.001**	0.038	0.003	0.640	0.500	0.199	**<0.001**	0.040	0.002	0.673	0.651	0.167	**<0.001**
relV̇O_2max_ (mL·kg·min)	−1.025	0.047	−0.782	0.612	3.828	11.6	**<0.001**	−0.920	0.039	−0.702	0.764	0.299	**<0.001**	−0.955	0.030	−0.730	0.858	2.336	**<0.001**
Grip strength (kg)	0.113	0.215	0.030	0.001	17.514	0.5	0.525	-	-	-	-	-	-	-	-	-	-	-	-
Right grip strength (kg)	0.032	0.113	0.016	0.000	9.157	0.1	0.780	-	-	-	-	-	-	-	-	-	-	-	-
Left grip strength (kg)	0.081	0.110	0.043	0.002	8.929	0.8	0.459	-	-	-	-	-	-	-	-	-	-	-	-
Leg strength (kg)	0.800	0.352	0.130	0.017	28.632	4.3	**0.024**	1.705	0.319	0.277	0.289	24.432	**<0.001**	1.786	0.306	0.290	0.340	23.426	**<0.001**
Push-ups (rpm)	−0.860	0.162	−0.293	0.086	13.178	11.5	**<0.001**	−0.433	0.155	−0.147	0.261	11.178	**0.006**	−0.449	0.155	−0.153	0.267	11.888	**0.004**
Sit-ups (rpm)	−0.832	0.119	−0.375	0.140	9.317	−11.0	**<0.001**	−0.563	0.116	−0.254	0.267	8.908	**<0.001**	−0.560	0.117	−0.252	0.268	8.950	**<0.001**
Sit-and-reach (cm)	−0.489	0.109	−0.250	0.062	8.872	−17.7	**<0.001**	−0.445	0.113	−0.227	0.113	8.657	**<0.001**	−0.445	0.112	−0.233	0.128	8.606	**<0.001**
Lean body Mass (kg)	0.681	0.113	0.333	0.106	9.302	20.7	**<0.001**	0.818	0.094	0.389	0.464	7.122	**<0.001**	0.917	0.070	0.436	0.708	5.245	**<0.001**
																			
Exploratory variable: body fat percentage																			
abV̇O_2max_ (L·min)	0.006	0.002	0.211	0.044	0.270	3.8	**<0.001**	0.015	0.002	0.507	0.343	0.225	**<0.001**	0.016	0.001	0.569	0.496	0.199	**0.014**
relV̇O_2max_ (mL·kg·min)	−0.315	0.032	−0.497	0.247	5.301	25.4	**<0.001**	−0.365	0.027	−0.575	0.588	3.936	**<0.001**	−0.400	0.024	−0.631	0.688	3.440	**<0.001**
Grip strength (kg)	−0.310	0.103	−0.170	0.029	17.251	2.3	**0.003**	0.178	0.104	0.098	0.271	15.000	0.089	-	-	-	-	-	-
Right grip strength (kg)	−0.168	0.054	−0.177	0.031	9.002	3.0	**0.002**	0.068	0.056	0.072	0.240	7.999	0.220	-	-	-	-	-	-
Left grip strength (kg)	−0.142	0.053	−0.153	0.023	8.831	1.6	**0.008**	0.109	0.053	0.118	0.269	7.668	**0.041**	0.148	0.051	0.160	0.342	7.307	**0.004**
Leg strength (kg)	−0.488	0.171	−0.163	0.027	28.536	7.1	**0.005**	0.319	0.176	0.106	0.235	25.378	0.072	-	-	-	-	-	-
Push-ups (rpm)	−0.464	0.078	−0.324	0.105	13.056	−15.7	**<0.001**	−0.306	0.082	−0.214	0.257	11.789	**<0.001**	−0.326	0.083	−0.228	0.283	11.769	**<0.001**
Sit-ups (rpm)	−0.434	0.057	−0.401	0.161	9.511	−14.1	**<0.001**	−0.369	0.061	−0.341	0.295	8.743	**<0.001**	−0.370	0.062	−0.341	0.296	8.785	**<0.001**
Sit-and-reach (cm)	−0.138	0.054	−0.145	0.021	9.036	−15.1	**0.011**	−0.214	0.060	−0.226	0.112	8.645	**<0.001**	−0.227	0.060	−0.240	0.128	8.581	**<0.001**
Lean body Mass (kg)	−0.151	0.057	−0.150	0.028	9.682	2.6	**0.003**	0.063	0.055	0.062	0.329	7.957	0.256	-	-	-	-	-	-

**Note:** Bold indicates statistical significance <0.05. CVHI—cardiovascular health index; V̇O_2max—_oxygen consumption; L·min—litres per min; mL·kg·min—millilitres per kilogram per minute; mmol·L—millimole per litre; mmHg—millimetres mercury; kg—kilogram; cm—centimetres; rpm—repetitions per minute; a—univariable linear regression; b—multivariable analysis; c—adjusted for age and sex; d—covariates adjusted for age, sex, weekly METs and height.; B—unstandardised beta coefficients; β—standardised beta coefficient; SE—standard error; R^2^—R squared; RMSE—root mean square error; CV—cross-validation; ^▲^—percentage difference for R^2^ between training and testing data.

**Table 5:** Multinomial logistic regression indicating the association between physical fitness and cardiovascular health index categories in firefighters.

	Univariable Model ^a^				Multivariable Model ^b^							
	Model 1				Model 2 ^c^				Model 3 ^d^			
	Intermediate CVHI		Good CVHI		Intermediate CVHI		Good CVHI		Intermediate CVHI		Good CVHI	
Model: CVHI (Poor CVHI)	OR (95% CI)	*p*	OR (95% CI)	*p*	OR (95% CI)	*p*	OR (95% CI)	*p*	OR (95% CI)	*p*	OR (95% CI)	*p*
abV̇O_2max_ (L·min)	0.980 (0.41, 2.35)	0.719	0.34 (0.09, 1.37)	0.131	-	-	0.09 (0.02, 0.51)	**0.006**	-	-	-	-
relV̇O_2max_ (mL·kg·min)	1.14 (1.09, 1.20)	**<0.001**	1.41 (1.29, 1.55)	**<0.001**	1.14 (1.08, 1.21)	**<0.001**	1.38 (1.24, 1.53)	**<0.001**	1.19 (1.11, 1.28)	**<0.001**	1.49 (1.33, 1.69)	**<0.001**
Grip strength (kg)	1.00 (0.99, 1.02)	0.602	0.97 (0.95, 0.99)	**0.009**	1.01 (0.99, 1.03)	0.687	0.98 (0.95, 1.01)	0.135	-	-	-	-
Right grip strength (kg)	1.01 (0.98, 1.03)	0.704	0.95 (0.91, 0.99)	**0.025**	1.01 (0.98, 1.05)	0.793	0.98 (0.92, 1.03)	0.271	-	-	-	-
Left grip strength (kg)	1.01 (0.98, 1.04)	0.390	0.94 (0.89, 0.98)	**0.006**	1.02 (0.98, 1.05)	0.608	0.95 (0.89, 1.01)	**0.074**	-	-	-	-
Leg strength (kg)	0.99 (0.99, 1.01)	0.501	0.99 (0.98, 1.01)	0.243	-	-	-	-	-	-	-	-
Push-ups (rpm)	1.04 (1.02, 1.06)	**<0.001**	1.05 (1.02, 1.08)	**0.003**	1.03 (1.01, 1.05)	**0.010**	1.03 (0.99, 1.07)	0.134	-	-	-	-
Sit-ups (rpm)	1.05 (1.03, 1.08)	**<0.001**	1.08 (1.03, 1.12)	**<0.001**	1.04 (1.01, 1.07)	**0.004**	1.06 (1.01, 1.11)	**0.023**	1.04 (1.01, 1.07)	**0.003**	1.06 (1.01, 1.11)	**0.023**
Sit-and-reach	1.04 (1.01, 1.07)	**0.004**	1.06 (1.01, 1.11)	**0.012**	1.04 (1.01, 1.07)	**0.020**	1.03 (0.98, 1.08)	0.236	1.04 (1.01, 1.07)	**0.016**	-	-

Note: Bold indicates statistical significance <0.05. CVHI—cardiovascular health index; V̇O_2max_—oxygen consumption; L·min—litres per min; mL·kg·min—millilitres per kilogram per minute; kg—kilogram; cm—centimetres; rpm—repetitions per minute; a—unadjusted univariable multinomial regression; b—multivariable model adjusted for age and sex; c—adjusted for age and sex; d—covariates adjusted for age, sex and weekly METs; OR—odds ratio, CI—confidence interval; *p*—significance level.

**Table 6:** Linear regression indicating the association between physical fitness and heart rate variability in firefighters.

		Univariable Linear Models ^a^						Multivariable Linear Models ^b^											
		Model 1						Model 2 ^c^						Model 3 ^d^					
	B	SE	β	R^2^	RMSE	CV ^▲^	*p*-Value	B	SE	β	R^2^	RMSE	*p*-Value	B	SE	β	R^2^	RMSE	*p*-Value
Dependent variable: Heart rate variability																			
abV̇O_2max_ (L·min)	269.929	25.75	0.517	0.268	125.078	5.4	**<0.001**	308.020	27.113	0.590	0.303	122.397	**<0.001**	317.504	29.285	0.608	0.316	125.283	**<0.001**
relV̇O_2max_ (mL·kg·min)	7.312	1.31	0.306	0.093	135.329	−6.3	**0.001**	11.136	1.557	0.466	0.148	135.328	**<0.001**	13.513	1.551	0.565	0.240	121.839	**<0.001**
Grip strength (kg)	0.498	0.482	0.060	0.04	145.633	−5.6	0.302	-	-	-	-		-	-	-	-	-	-	-
Right grip strength (kg)	−0.092	0.293	0.023	0.00	145.859	−2.6	0.755	-	-	-	-		-	-	-	-	-	-	-
Left grip strength (kg)	1.178	0.611	0.094	0.012	145.234	−8.5	0.055	-	-	-	-		-	-	-	-	-	-	-
Leg strength (kg)	1.366	0.816	−0.018	0.009	145.929	0.5	0.095	-	-	-	-		-	-	-	-	-	-	-
Push-ups (rpm)	1.486	0.922	0.112	0.009	144.757	−15.6	0.108	-	-	-	-		-	-	-	-	-	-	-
Sit-ups (rpm)	1.085	0.865	0.097	0.005	145.150	−6.9	0.211	-	-	-	-		-	-	-	-	-	-	-
																			
Dependent variable: SDNN																			
abV̇O_2max_ (L·min)	40.363	3.797	0.522	0.273	18.445	21.7	**<0.001**	36.076	4.023	0.467	0.299	18.178	**<0.001**	37.626	4.312	0.487	0.321	17.954	**<0.001**
relV̇O_2max_ (mL·kg·min)	1.685	0.179	0.478	0.228	19.003	−18.8	**<0.001**	1.594	0.216	0.452	0.247	18.834	**<0.001**	1.838	0.217	0.522	0.302	18.057	**<0.001**
Grip strength (kg)	0.179	0.071	0.144	0.021	21.439	−0.2	**0.039**	0.154	0.079	0.124	0.116	20.445	0.053	-	-	-	-	-	-
Right grip strength (kg)	0.120	0.043	0.114	0.026	21.525	−0.2	**0.036**	0.198	0.150	0.083	0.109	20.515	0.187	-	-	-	-	-	-
Left grip strength (kg)	0.455	0.087	0.166	0.084	21.365	−4.2	**<0.001**	0.373	0.154	0.154	0.112	20.373	**0.016**	0.325	0.159	0.134	0.155	20.060	**0.042**
Leg strength (kg)	0.525	0.118	0.160	0.25	21.346	−2.1	**<0.001**	0.068	0.047	0.090	0.110	20.467	0.148	-	-	-	-	-	-
Push-ups (rpm)	0.455	0.087	0.291	0.84	20.658	−1.9	**<0.001**	0.266	0.098	0.170	0.128	20.212	**0.007**	0.251	0.097	0.161	0.166	-	**0.010**
Sit-ups (rpm)	0.525	0.118	0.250	0.063	24.533	5.7	**0.013**	0.271	0.130	0.129	0.116	24.274	**0.007**	0.270	0.127	0.129	0.142	20.082	**0.035**
																			
Dependent variable: RMSSD																			
abV̇O_2max_ (L·min)	46.006	4.486	0.508	0.259	21.795	19.6	**<0.001**	43.592	4.816	0.482	0.269	21.719	**<0.001**	46.728	5.124	0.516	0.301	21.834	**<0.001**
relV̇O_2max_ (mL·kg·min)	1.956	0.209	0.473	0.225	22.294	−10.6	**<0.001**	2.049	0.255	0.516	0.234	22.239	**<0.001**	2.307	0.257	0.559	0.296	22.408	**<0.001**
Grip strength (kg)	0.115	0.084	508	0.006	25.267	−1.3	0.173	-	-	-	-		-	-	-	-	-	-	-
Right grip strength (kg)	0.068	0.051	0.022	0.006	25.317	−0.3	0.184	-	-	-	-		-	-	-	-	-	-	-
Left grip strength (kg)	0.432	0.104	0.106	0.056	25.205	−2.4	**<0.001**	0.278	0.185	0.098	0.070	24.521	0.134	-	-	-	-	-	-
Leg strength (kg)	0.447	0.14	0.077	0.033	25.235	−3.4	**0.002**	0.015	0.056	0.017	0.063	24.579	0.788	-	-	-	-	-	-
Push-ups (rpm)	0.441	0.159	0.235	0.025	24.533	−14.9	**<0.001**	0.266	0.118	0.016	0.082	24.274	**0.025**	0.243	0.116	0.133	0.118	23.893	**0.037**
Sit-ups (rpm)	−0.066	0.151	0.182	0.001	24.958	−6.0	0.664	-	-	-	-		-	-	-	-	-	-	-
																			
Dependent variable: low-frequency																			
abV̇O_2max_ (L·min)	0.011	0.005	0.123	0.123	0.026	0.5	**0.032**	0.001	0.005	0.016	0.087	0.025	0.792	-	-	-	-	-	-
relV̇O_2max_ (mL·kg·min)	0.001	0.000	0.217	0.047	0.025	6.7	**<0.001**	0.001	0.000	0.124	0.097	0.025	0.065	-	-	-	-	-	-
Grip strength (kg)	0.000	0.000	0.070	0.005	0.026	−2.1	0.227	-	-	-	-		-	-	-	-	-	-	-
Right grip strength (kg)	0.000	0.000	0.081	0.042	0.026	−2.8	0.160	-	-	-	-		-	-	-	-	-	-	-
Left grip strength (kg)	0.000	0.000	0.054	0.05	0.026	−1.3	0.353	-	-	-	-		-	-	-	-	-	-	-
Leg strength (kg)	0.000	0.000	0.204	0.022	0.025	−7.2	**<0.001**	0.000	0.000	0.127	0.094	0.025	**0.041**	0.000	0.000	0.118	0.101	0.025	0.073
Push-ups (rpm)	0.000	0.000	0.225	0.005	0.025	−6.0	0.236	-	-	-	-		-	-	-	-	-	-	-
Sit-ups (rpm)	4.332	0.000	0.147	0.000	0.025	−0.6	0.977	-	-	-	-		-	-	-	-	-	-	-
																			
Dependent variable: high-frequency																			
abV̇O_2max_ (L·min)	−0.015	0.013	−0.066	0.004	0.063	−2.6	0.251	-	-	-	-		-	-	-	-	-		-
relV̇O_2max_ (mL·kg·min)	0.001	0.001	0.122	0.015	0.062	8.7	**0.033**	0.002	0.001	0.163	0.032	0.062	**0.019**	0.002	0.001	0.163	0.042	0.062	**0.027**
Grip strength (kg)	−0.001	0.000	−0.152	0.023	0.062	−0.5	**0.008**	0.000	0.000	−0.125	0.026	0.062	0.066	-	-	-	-	-	-
Right grip strength (kg)	0.000	0.000	−0.154	0.031	0.062	0.6	**0.002**	−0.001	0.000	−0.126	0.027	0.062	0.057	-	-	-	-	-	-
Left grip strength (kg)	−2.858	0.000	−0.141	0.000	0.062	−1.5	0.914	-	-	-	-		-	-	-	-	-	-	-
Leg strength (kg)	0.000	0.000	−0.177	0.031	0.061	1.8	**0.002**	0.000	0.000	−0.163	0.035	0.062	**0.014**	0.000	0.000	−0.192	0.056	0.062	**0.005**
Push-ups (rpm)	0.000	0.000	−0.005	0.002	0.062	−5.6	0.495	-	-	-	-		-	-	-	-	-	-	-
Sit-ups (rpm)	−0.001	0.000	0.015	0.022	0.062	−14.5	**<0.001**	0.000	0.000	0.031	0.016	0.062	0.640	-	-	-	-	-	-
																			
Dependent variable: low/high-frequency ratio																			
abV̇O_2max_ (L·min)	−2.914	0.862	−0.191	0.037	4.159	−0.3	**<0.001**	−3.045	0.923	−0.200	0.055	4.133	**0.001**	−3.880	0.992	−0.254	0.083	4.092	**<0.001**
relV̇O_2max_ (mL·kg·min)	−0.191	0.038	−0.276	0.076	4.073	6.4	**<0.001**	−0.210	0.047	−0.305	0.083	4.072	**<0.001**	−0.209	0.049	−0.303	0.092	4.072	**<0.001**
Grip strength (kg)	0.016	0.014	0.064	0.004	4.243	0.7	0.265	-	-	-	-		-	-	-	-	-	-	-
Right grip strength (kg)	0.010	0.009	0.068	0.004	4.240	0.5	0.265	-	-	-	-		-	-	-	-	-	-	-
Left grip strength (kg)	0.027	0.018	0.056	0.015	4.244	0.6	0.332	-	-	-	-		-	-	-	-	-	-	-
Leg strength (kg)	−0.014	0.024	0.065	0.001	4.247	0.4	0.565	-	-	-	-		-	-	-	-	-	-	-
Push-ups (rpm)	−0.038	0.018	−0.123	0.015	4.221	−6.1	**0.034**	−0.035	0.021	−0.114	0.029	4.205	0.086	-	-	-	-	-	-
Sit-ups (rpm)	−0.014	0.024	−0.033	0.001	4.217	−5.9	0.565	-	-	-	-		-	-	-	-	-	-	-

Note: Bold indicates statistical significance <0.05. CVHI—cardiovascular health index; V̇O_2max_—oxygen consumption; L·min—litres per min; mL·kg·min—millilitres per kilogram per minute; mmol·L—millimole per litre; mmHg—millimetres mercury; kg—kilogram; cm—centimetres; rpm—repetitions per minute; SDNN—standard deviation of N-N intervals; RMSSD—root mean square of successive differences between normal heartbeats; a—univariable linear regression; b—multivariable analysis; c—adjusted for age and sex; d—covariates adjusted for age, sex, weekly METs and height. B—unstandardised beta coefficients; β—standardised beta coefficient; SE—standard error; R^2^—R squared; RMSE—root mean square error; CV—cross-validation; ^▲^—percentage difference for R^2^ between training and testing data.

**Table 7:** LASSO-derived multivariable linear regression coefficients to discern key physical fitness parameters most associated with CVH in firefighters.

	LASSO Coefficients										
	Physical Fitness										
	Prediction	Estimate	RMSE	abV̇O_2max_	relV̇O_2max_	GS	LS	PU	SU	SaR	LBM
Systolic blood pressure	0.905	0.923	0.941	-	−0.086	-	-	-	-	-	0.085
Diastolic blood pressure	0.903	0.930	0.951	-	−0.172	-	-	-	−0.007	-	-
Non-fasting blood glucose	0.954	0.969	0.978	−0.001	−0.100	-	-	-	-	-	-
Total cholesterol	0.965	0.602	0.986	-	−0.064	-	-	-	-	-	-
Low-density lipoprotein cholesterol	0.989	0.996	1.000	-	0.004	-	-	-	-	-	-
High-density lipoprotein cholesterol	0.894	0.930	0.804	−0.062	0.148		−0.134	0.067	-	-	-
Triglycerides	0.871	0.901	0.936	-	−0.184	-	-	-	-	-	-
MET minutes	0.993	0.999	1.000	-	-	-	0.007	-	-	-	-
Cardiovascular health index	0.942	0.958	0.992	-	0.64	-		-	-	-	-
											
HRV	0.635	0.649	0.791	0.437	0.213	-	−0.003	-	-	-	-
SDNN	0.476	0.479	0.686	0.457	0.397	-	-	-	-	-	-
RMSSD	0.518	0.527	0.715	0.438	0.372	-	-	-	-	-	-
LF	0.984	0.998	0.987	-	-	-	0.013	0.028	-	-	-
HF	0.995	0.999	0.994	-	-	-	−0.004	-	-	-	-
LF/HF ratio	0.911	0.943	0.948	−0.089	−0.150	-	-	-	-	-	-

Note: RMSE—root-mean-square error; abCRF—absolute cardiorespiratory fitness; relCRF—relative cardiorespiratory fitness; GS—grip strength; LS—leg strength; PU—push-ups; SU—sit-ups; SaR—sit-and-reach; LBM—lean body mass; “-“—indicates coefficients of zero; HRV—heart rate variability; SDNN—standard deviation of N-N intervals; RMSSD—root mean square of successive differences between normal heartbeats; LF—low frequency; HF—high frequency; LF/HF ratio—low-frequency/high-frequency ratio.

## Data Availability

The data presented in this study are available on request from the corresponding author. The data are not publicly available due to this being an ongoing study and the POPIA Act, which restricts access to private information being made publicly available.

## References

[1] Smith D.L., Haller J.M., Korre M., Sampani K., Porto L.G.G., Fehling P.C., Christophi C.A., Kales S.N. (2019). The Relation of Emergency Duties to Cardiac Death Among US Firefighters. Am. J. Cardiol..

[2] Chizewski A., Box A., Kesler R., Petruzzello S.J. (2021). Fitness Fights Fires: Exploring the Relationship between Physical Fitness and Firefighter Ability. Int. J. Environ. Res. Public Health.

[3] Park K., Rosengren K.S., Horn G.P., Smith D.L., Hsiao-Wecksler E.T. (2011). Assessing Gait Changes in Firefighters Due to Fatigue and Protective Clothing. Saf. Sci..

[4] Taylor N.A.S., Lewis M.C., Notley S.R., Peoples G.E. (2012). A Fractionation of the Physiological Burden of the Personal Protective Equipment Worn by Firefighters. Eur. J. Appl. Physiol..

[5] Henderson N., Berry M., Matic T. (2007). Field Measures of Strength and Fitness Predict Firefighter Performance on Physically Demanding Tasks. Pers. Psychol..

[6] Sothmann M.S., Gebhardt D.L., Baker T.A., Kastellos G.M., Sheppard V.A. (2004). Performance Requirements of Physically Strenuous Occupations: Validating Minimum Standards for Muscular Strength and Endurance. Ergonomics.

[7] Baur D.M., Christophi C.A., Tsismenakis A.J., Cook E.F., Kales S.N. (2011). Cardiorespiratory Fitness Predicts Cardiovascular Risk Profiles in Career Firefighters. J. Occup. Environ. Med..

[8] Smith D.L., Haller J.M., Korre M., Fehling P.C., Sampani K., Grossi Porto L.G., Christophi C.A., Kales S.N. (2018). Pathoanatomic Findings Associated with Duty-Related Cardiac Death in US Firefighters: A Case–Control Study. J. Am. Heart Assoc..

[9] Dorman L.E., Havenith G. (2009). The Effects of Protective Clothing on Energy Consumption during Different Activities. Eur. J. Appl. Physiol..

[10] Elsner K.L., Kolkhorst F.W. (2008). Metabolic Demands of Simulated Firefighting Tasks. Ergonomics.

[11] Frost D.M., Beach T.A.C., Crosby I., McGill S.M. (2016). The Cost and Distribution of Firefighter Injuries in a Large Canadian Fire Department. Work.

[12] von Heimburg E.D., Rasmussen A.K.R., Medbø J.I. (2006). Physiological Responses of Firefighters and Performance Predictors during a Simulated Rescue of Hospital Patients. Ergonomics.

[13] Ras J., Kengne A.P., Smith D., Soteriades E.S., Leach L. (2022). Effects of Cardiovascular Health, Musculoskeletal Health and Physical Fitness on Occupational Performance of Firefighters: Protocol for a Systematic Review and Meta-Analysis. BMJ Open.

[14] Rhea M.R., Alvar B.A., Gray R. (2004). Physical Fitness and Job Performance of Firefighters. J. Strength Cond. Res..

[15] Michaelides M.A., Parpa K.M., Thompson J., Brown B. (2008). Predicting Performance on a Firefghter’s Ability Test from Fitness Parameters. Res. Q. Exerc. Sport.

[16] Yu C.C.W., Au C.T., Lee F.Y.F., So R.C.H., Wong J.P.S., Mak G.Y.K., Chien E.P., McManus A.M. (2015). Association between Leisure Time Physical Activity, Cardiopulmonary Fitness, Cardiovascular Risk Factors, and Cardiovascular Workload at Work in Firefighters. Saf. Health Work.

[17] Mehrdad R., Movasatian F., Momenzadeh A.S. (2013). Fitness for Work Evaluation of Firefighters in Tehran. Acta Med. Iran..

[18] Ras J., Mosie D., Strauss M., Leach L. (2021). Knowledge of and Attitudes toward Health and Cardiovascular Disease Risk Factors among Firefighters in Cape Town, South Africa. J. Public Health Res..

[19] Muegge C.M., Zollinger T.W., Song Y., Wessel J., Monahan P.O., Moffatt S.M. (2020). Barriers to Weight Management among Overweight and Obese Firefighters. J. Occup. Environ. Med..

[20] Ras J., Leach L. (2022). Firefighters’ Health Knowledge, Cardiovascular Disease Risk Factors and Sociodemographic Characteristics as Predictors of Firefighters Attitudes Toward Health. J. Occup. Environ. Med..

[21] Dobson M., Choi B., Schnall P.L., Wigger E., Garcia-Rivas J., Israel L., Baker D.B. (2013). Exploring Occupational and Health Behavioral Causes of Firefighter Obesity: A Qualitative Study. Am. J. Ind. Med..

[22] Kirlin L.K., Nichols J.F., Rusk K., Parker R.A., Rauh M.J. (2017). The Effect of Age on Fitness among Female Firefighters. Occup. Med..

[23] Kiss P., de Meester M., Maes C., de Vriese S., Kruse A., Braeckman L. (2014). Cardiorespiratory Fitness in a Representative Sample of Belgian Firefighters. Occup. Med..

[24] Walker A., Driller M., Argus C., Cooke J., Rattray B. (2014). The Ageing Australian Firefighter: An Argument for Age-Based Recruitment and Fitness Standards for Urban Fire Services. Ergonomics.

[25] Perroni F., Guidetti L., Cignitti L., Baldari C. (2015). Age-Related Changes in Upper Body Strength and Lower Limb Power of Professional Italian Firefighters. Sport Sci. Health.

[26] Ras J., Leach L. (2021). Prevalence of Coronary Artery Disease Risk Factors in Firefighters in the City of Cape Town Fire and Rescue Service—A Descriptive Study. J. Public Health Res..

[27] Martin Z.T., Schlaff R.A., Hemenway J.K., Coulter J.R., Knous J.L., Lowry J.E., Ode J.J. (2019). Cardiovascular Disease Risk Factors and Physical Fitness in Volunteer Firefighters. Int. J. Exerc. Sci..

[28] Savall A., Charles R., Bertholon A., Gramont B., Trombert B., Barthélémy J.C., Roche F. (2020). Volunteer and Career French Firefighters: Cardiovascular Risk Factors and Cardiovascular Risk Assessment. Eur. J. Prev. Cardiol..

[29] Baur D.M., Christophi C.A., Cook E.F., Kales S.N. (2012). Age-Related Decline in Cardiorespiratory Fitness among Career Firefighters: Modification by Physical Activity and Adiposity. J. Obes..

[30] Seyedmehdi S.M., Attarchi M., Cherati A.S., Hajsadeghi S., Tofighi R., Jamaati H. (2016). Relationship of Aerobic Fitness with Cardiovascular Risk Factors in Firefighters. Work.

[31] Strauss M., Foshag P., Jehn U., Brzęk A., Littwitz H., Leischik R. (2021). Higher Cardiorespiratory Fitness Is Strongly Associated with Lower Cardiovascular Risk Factors in Firefighters : A Cross-Sectional Study in a German Fire Brigade. Sci. Rep..

[32] Espinoza F., Delgado-Floody P., Martínez-Salazar C., Jerez-Mayorga D., Guzmán-Guzmán I.P., Caamaño-Navarrete F., Ramirez-Campillo R., Chamorro C., Campos-Jara C. (2019). The Influence of Cardiometabolic Risk Factors on Cardiorespiratory Fitness in Volunteer Chilean Firefighters. Am. J. Hum. Biol..

[33] McAllister M.J., Gonzalez D.E., Leonard M., Martaindale M.H., Bloomer R.J., Pence J., Martin S.E. (2022). Firefighters with Higher Cardiorespiratory Fitness Demonstrate Lower Markers of Cardiovascular Disease Risk. J. Occup. Environ. Med..

[34] Durand G., Tsismenakis A.J., Jahnke S.A., Baur D.M., Christophi C.A., Kales S.N. (2011). Firefighters’ Physical Activity: Relation to Fitness and Cardiovascular Disease Risk. Med. Sci. Sport. Exerc..

[35] Ras J., Smith D.L., Kengne A.P., Soteriades E.E., Leach L. (2022). Cardiovascular Disease Risk Factors, Musculoskeletal Health, Physical Fitness, and Occupational Performance in Firefighters: A Narrative Review. J. Environ. Public Health.

[36] Ras J., Leach L. (2022). Relationship between Physical Activity, Coronary Artery Disease Risk Factors and Musculoskeletal Injuries in the City of Cape Town Fire and Rescue Service. INQUIRY J. Health Care Organ. Provis. Financ..

[37] Bode E.D., Mathias K.C., Stewart D.F., Moffatt S.M., Jack K., Smith D.L. (2021). Cardiovascular Disease Risk Factors by BMI and Age in United States Firefighters. Obesity.

[38] Ras J., Smith D.L., Soteriades E.S., Kengne A.P., Leach L. (2022). A Pilot Study on the Relationship between Cardiovascular Health, Musculoskeletal Health, Physical Fitness and Occupational Performance in Firefighters. Eur. J. Investig. Health Psychol. Educ..

[39] Liguori G., Medicine A.C., Fountaine C.J. (2021). ACSM’s Guidelines for Exercise Testing and Prescription.

[40] Bianco A., Lupo C., Alesi M., Spina S., Raccuglia M., Thomas E., Paoli A., Palma A. (2015). The Sit up Test to Exhaustion as a Test for Muscular Endurance Evaluation. Springerplus.

[41] Lloyd-Jones D.M., Hong Y., Labarthe D., Mozaffarian D., Appel L.J., van Horn L., Greenlund K., Daniels S., Nichol G., Tomaselli G.F. (2010). Defining and Setting National Goals for Cardiovascular Health Promotion and Disease Reduction. Circulation.

[42] Kim S., Chang Y., Cho J., Hong Y.S., Zhao D., Kang J., Jung H.-S., Yun K.E., Guallar E., Ryu S. (2019). Life’s Simple 7 Cardiovascular Health Metrics and Progression of Coronary Artery Calcium in a Low-Risk Population. Arter. Thromb. Vasc. Biol..

[43] Ketelaar E.J., Vos A.G., Godijk N.G., Scheuermaier K., Devillé W., Tempelman H., Coutinho R.A., Venter W.D.F., Grobbee D.E., Klipstein-Grobusch K. (2020). Ideal Cardiovascular Health Index and Its Determinants in a Rural South African Population. Glob. Heart.

[44] Lloyd-Jones D.M., Leip E.P., Larson M.G., D’Agostino R.B., Beiser A., Wilson P.W.F., Wolf P.A., Levy D. (2006). Prediction of Lifetime Risk for Cardiovascular Disease by Risk Factor Burden at 50 Years of Age. Circulation.

[45] Jones N.R., McCormack T., Constanti M., McManus R.J. (2020). Diagnosis and Management of Hypertension in Adults: NICE Guideline Update 2019. Br. J. Gen. Pract..

[46] Kaleta D., Makowiec-Dąbrowska T., Dziankowska-Zaborszczyk E., Fronczak A. (2012). Determinants of Heavy Smoking: Results from the Global Adult Tobacco Survey in Poland (2009–2010). Int. J. Occup. Med. Environ. Health.

[47] D’Agostino R.B., Vasan R.S., Pencina M.J., Wolf P.A., Cobain M., Massaro J.M., Kannel W.B. (2008). General Cardiovascular Risk Profile for Use in Primary Care. Circulation.

[48] Arnett D.K., Blumenthal R.S., Albert M.A., Buroker A.B., Goldberger Z.D., Hahn E.J., Himmelfarb C.D., Khera A., Lloyd-Jones D., McEvoy J.W. (2019). 2019 ACC/AHA Guideline on the Primary Prevention of Cardiovascular Disease: A Report of the American College of Cardiology/American Heart Association Task Force on Clinical Practice Guidelines. Circulation.

[49] Billman G.E., Huikuri H.V., Sacha J., Trimmel K. (2015). An Introduction to Heart Rate Variability: Methodological Considerations and Clinical Applications. Front. Physiol..

[50] Tomes C., Schram B., Orr R. (2020). Relationships between Heart Rate Variability, Occupational Performance, and Fitness for Tactical Personnel: A Systematic Review. Front. Public Health.

[51] Vaara J.P., Fogelholm M., Vasankari T., Santtila M., Häkkinen K., Kyröläinen H. (2014). Associations of Maximal Strength and Muscular Endurance with Cardiovascular Risk Factors. Int. J. Sport. Med..

[52] Shin J.Y., Ha C.H. (2016). Relationships between Blood Pressure and Health and Fitness-Related Variables in Obese Women. J. Phys. Ther. Sci..

[53] Lin G.-M., Tsai K.-Z., Lin C.-S., Han C.-L. (2019). Physical Fitness and Long-Term Blood Pressure Variability in Young Male Military Personnel. Curr. Hypertens. Rev..

[54] Gubelmann C., Vollenweider P., Marques-Vidal P. (2017). Association of Grip Strength with Cardiovascular Risk Markers. Eur. J. Prev. Cardiol..

[55] Yang J., Christophi C.A., Farioli A., Baur D.M., Moffatt S. (2019). Association between Push-up Exercise Capacity and Future Cardiovascular Events among Active Adult Men. JAMA Netw. Open.

[56] Ras J., Kengne A.P., Smith D.L., Soteriades E.S., Leach L. (2023). Association between Cardiovascular Disease Risk Factors and Cardiorespiratory Fitness in Firefighters: A Systematic Review and Meta-Analysis. Int. J. Environ. Res. Public Health.

[57] DeFina L.F., Haskell W.L., Willis B.L., Barlow C.E., Finley C.E., Levine B.D., Cooper K.H. (2015). Physical Activity Versus Cardiorespiratory Fitness: Two (Partly) Distinct Components of Cardiovascular Health?. Prog. Cardiovasc. Dis..

[58] Yamada E., Takeuchi M., Kurata M., Tsuboi A., Kazumi T., Fukuo K. (2015). Low Haemoglobin Levels Contribute to Low Grip Strength Independent of Low-Grade Inflammation in Japanese Elderly Women. Asia Pac. J. Clin. Nutr..

[59] Carter J.R., Ray C.A., Downs E.M., Cooke W.H. (2003). Strength Training Reduces Arterial Blood Pressure but Not Sympathetic Neural Activity in Young Normotensive Subjects. J. Appl. Physiol..

[60] Martel G.F., Hurlbut D.E., Lott M.E., Lemmer J.T., Ivey F.M., Roth S.M., Rogers M.A., Fleg J.L., Hurley B.F. (1999). Strength Training Normalizes Resting Blood Pressure in 65- to 73-Year-Old Men and Women with High Normal Blood Pressure. J. Am. Geriatr. Soc..

[61] Singh N., Liu K., Tian L., Criqui M.H., Guralnik J.M., Ferrucci L., Liao Y., McDermott M.M. (2010). Leg Strength Predicts Mortality in Men but Not in Women with Peripheral Arterial Disease. J. Vasc. Surg..

[62] Taylor P., Beach T.A.C., Frost D.M., Mcgill S.M., Callaghan J.P. (2014). Physical Fitness Improvements and Occupational Low- Back Loading—An Exercise Intervention Study with Firefighters. Ergonomics.

[63] Andrews K.L., Gallagher S., Herring M.P. (2019). The Effects of Exercise Interventions on Health and Fitness of Firefighters: A Meta-Analysis. Scand. J. Med. Sci. Sport..

[64] Findley B.W., Brown L.E., Whitehurst M., Gilbert R., Apold S.A. (1995). Age-Group Performance and Physical Fitness in Male Firefighters. J. Strength Cond. Res..

[65] Nowak A., Molik B., Wójcik A., Rutkowska I., Nowacka-Dobosz S., Kowalczyk M., Marszałek J. (2018). Physical Activity and Injuries Relating to Physical Fitness of Professional Firefighters. Adv. Rehabil..

[66] Perroni F., Cignitti L., Cortis C., Capranica L. (2014). Physical Fitness Profile of Professional Italian Firefighters: Differences among Age Groups. Appl. Ergon..

[67] Gerstner G.R., Giuliani H.K., Mota J.A., Ryan E.D. (2018). Influence of Muscle Quality on the Differences in Strength from Slow to Fast Velocities in Career Firefighters. J. Strength Cond. Res..

[68] Álvarez C., Cadore E., Gaya A.R., Mello J.B., Reuter C.P., Delgado-Floody P., Ramos-Sepúlveda J.A., Carrillo H.A., Devia D.G., Ramírez-Vélez R. (2022). Associations of Cardiorespiratory Fitness and Obesity Parameters with Blood Pressure: Fitness and Fatness in Youth Latin-American Ethnic Minority. Ethn. Health.

[69] Macdonald H.V., Pescatello L.S. (2019). Cardiorespiratory Fitness in Cardiometabolic Diseases.

[70] Davison K., Bircher S., Hill A., Coates A.M., Howe P.R.C., Buckley J.D. (2010). Relationships between Obesity, Cardiorespiratory Fitness, and Cardiovascular Function. J. Obes..

[71] Poston W.S.C., Haddock C.K., Jahnke S.A., Jitnarin N., Tuley B.C., Kales S.N. (2011). The Prevalence of Overweight, Obesity, and Substandard Fitness in a Population-Based Firefighter Cohort. J. Occup. Environ. Med..

[72] Oktay A.A., Lavie C.J., Kokkinos P.F., Parto P., Pandey A., Ventura H.O. (2017). The Interaction of Cardiorespiratory Fitness with Obesity and the Obesity Paradox in Cardiovascular Disease. Prog. Cardiovasc. Dis..

[73] Mayer J.M., Nuzzo J.L., Chen R., Quillen W.S., Verna J.L., Miro R., Dagenais S. (2012). The Impact of Obesity on Back and Core Muscular Endurance in Firefighters. J. Obes..

[74] Porto L.G.G., Schmidt A.C.B., de Souza J.M., Nogueira R.M., Fontana K.E., Molina G.E., Korre M., Smith D.L., Junqueira L.F., Kales S.N. (2019). Firefighters’ Basal Cardiac Autonomic Function and Its Associations with Cardiorespiratory Fitness. Work.

[75] Lesniak A.Y., Sell K.M., Morris C., Abel M.G. (2022). Relationship between Heart Rate Variability vs. Occupational Performance, Physical Activity and Fitness Measures in Structural Firefighters. J. Sport Hum. Perform..

[76] Grant C.C., Mongwe L., Janse Van Rensburg D.C., Fletcher L., Wood P.S., Terblanche E., du Toit P.J. (2016). The Difference between Exercise-Induced Autonomic and Fitness Changes Measured after 12 and 20 Weeks of Medium-to-High Intensity Military Training. J. Strength Cond. Res..

[77] Ras J., Leach L., Mentor D. (2022). Use of Mobile Technology in Assessing Occupational Performance and Stress in Firefighters. Handbook of Research on New Media, Training, and Skill Development for the Modern Workforce.

